# Orderly progression through S-phase requires dynamic ubiquitylation and deubiquitylation of PCNA

**DOI:** 10.1038/srep25513

**Published:** 2016-05-06

**Authors:** Vanesa Álvarez, Laura Viñas, Alfonso Gallego-Sánchez, Sonia Andrés, María P. Sacristán, Avelino Bueno

**Affiliations:** 1 Instituto de Biología Molecular y Celular del Cáncer, Universidad de Salamanca/CSIC, 37007 Salamanca, Spain; 2 Departamento de Microbiología y Genética, Universidad de Salamanca, 37007 Salamanca, Spain

## Abstract

Proliferating-cell nuclear antigen (PCNA) is a DNA sliding clamp with an essential function in DNA replication and a key role in tolerance to DNA damage by ensuring the bypass of lesions. In eukaryotes, DNA damage tolerance is regulated by ubiquitylation of lysine 164 of PCNA through a well-known control mechanism; however, the regulation of PCNA deubiquitylation remains poorly understood. Our work is a systematic and functional study on PCNA deubiquitylating enzymes (DUBs) in *Schizosaccharomyces pombe.* Our study reveals that the deubiquitylation of PCNA in fission yeast cells is a complex process that requires several ubiquitin proteases dedicated to the deubiquitylation of a specific subnuclear fraction of mono- and di-ubiquitylated PCNA or a particular type of poly-ubiquitylated PCNA and that there is little redundancy among these enzymes. To understand how DUB activity regulates the oscillatory pattern of ubiquitylated PCNA in fission yeast, we assembled multiple DUB mutants and found that a quadruple mutation of *ubp2*^+^, *ubp12*^+^, *ubp15*^+^, and *ubp16*^+^ leads to the stable accumulation of mono-, di-, and poly-ubiquitylated forms of PCNA, increases S-phase duration, and sensitizes cells to DNA damage. Our data suggest that the dynamic ubiquitylation and deubiquitylation of PCNA occurs during S-phase to ensure processive DNA replication.

Living cells tolerate DNA damage during S-phase to prevent the risk of irreversible DNA replication fork collapse^1^. DNA damage tolerance is based on translesion synthesis (TLS), which involves a dual mechanism, primarily mediated by specialized low fidelity DNA polymerases called TLS-polymerases. These TLS polymerases can be mutagenic because they induce an error-prone process that causes mutations. The second mechanism depends on template switching, the error-free component of the bypass that involves sister-strand pairing between nascent chains within the same replication fork. It is well established that both mechanisms efficiently prevent replisome stalling at damaged sites. Eukaryotic cells regulate the choice of alternative pathways/mechanisms to bypass DNA lesions during S-phase through the ubiquitylation of proliferating-cell nuclear antigen (PCNA), a processivity factor for DNA synthesis^2^,^3^. The monoubiquitylation of PCNA (ubPCNA) at Lys164 enhances the affinity of error-prone TLS DNA polymerases, and further polyubiquitylation of Lys164-monoubiquitylated PCNA promotes template switching^2^,^3^,^4^,^5^,^6^. The evolutionarily conserved Lys164-ubiquitylation of PCNA and its crucial role in DNA damage tolerance are well understood^7^,^8^,^9^. However, little is known regarding the significance of PCNA deubiquitylation in eukaryotes.

Work from the past decade has identified mammalian Usp1, human Usp10, and budding yeast Ubp10 as major deubiquitylating enzymes (DUBs) for PCNA^1^,^10^,^11^,^12^. Usp1 has been identified as a DUB that deubiquitylates mono-ubPCNA and mono-ubFANCD2 in human cells^10^. Upon UV light-induced DNA damage, Usp1 undergoes autocleavage, and PCNA therefore becomes ubiquitylated, suggesting that Usp1 continuously deubiquitylates PCNA in the absence of DNA damage. However, ubPCNA accumulation does not correlate with Usp1 (auto-induced) proteolysis when cells are exposed to other genotoxic agents, such as MMS and mitomycin C^13^,^14^, or the DNA replication blocking agent HU^15^, suggesting either that another human PCNA DUB exists or that Usp1 activity is regulated in a different manner in response to other DNA damaging agents. Therefore, the relevance of USP1 in reverting PCNA ubiquitylation when confronted with different DNA-damaging agents remains unclear. This is an important issue, particularly because PCNA ubiquitylation is required for mammalian cell survival not only after UV irradiation but also upon exposure to HU and MMS^16^,^17^. However, consistent with a role as a PCNA DUB, the depletion of chicken *USP1* in DT40 cells or in murine *Usp1*^−/−^ MEFs increases PCNA (and also FANCD2) mono-ubiquitylation^14^,^18^. More recently, it has been shown that upon UV-mediated DNA damage, HeLa cells rely on USP10 to deubiquitylate ISGylated-PCNA^12^. We have previously shown that the ubiquitin protease Ubp10 deubiquitylates K164 mono-and di-ubiquitylated PCNA during S phase in the yeast *Saccharomyces cerevisiae*^11^. Furthermore, we demonstrated that Ubp10 forms a complex with PCNA *in vivo*, as expected for an enzyme-substrate complex. Additionally, in agreement with a direct role as a PCNA DUB, we found that only catalytically active Ubp10 reverts PCNA ubiquitylation. However, despite the identification of Usp1, Usp10, and Ubp10 as PCNA DUBs, little is known regarding the deubiquitylation of ubPCNA in any other model organisms.

In the fission yeast *S. pombe*, a key model organism in understanding the cell division cycle, the proteases that deubiquitylate ubPCNA remain unknown. Potential candidates in fission yeast are 20 genes that encode different deubiquitylating enzymes or DUBs including 14 ubiquitin-specific proteases (USPs), 2 ubiquitin C-terminal hydrolases (UCHs), 2 ovarian tumour proteases (OTUs) and 2 JAB1/MPN/Mov34 metalloenzymes (JAMMs)^19^. Here, we show that Ubp2, Ubp15, and Ubp16 ubiquitin proteases, likely with the help of Ubp12, revert PCNA ubiquitylation in the fission yeast *S. pombe* during S-phase. All the DUBs involved in PCNA deubiquitylation in this model system belong to the same subfamily of ubiquitin-specific proteases (USPs/UBPs). We found that each of these ubiquitin proteases is dedicated to the deubiquitylation of a specific subnuclear fraction or a particular type of ubiquitylated PCNA. Our data suggest that the dynamic ubiquitylation and deubiquitylation of PCNA occurs during S-phase and ensures proper DNA replication progression in *S. pombe*. Consequently, we propose that excessive DNA replication bypass interferes with the normal progression of DNA replication forks during S-phase.

## Results

### Analysis of PCNA deubiquitylation in the fission yeast *Schizosaccharomyces pombe*

**Figure 1:**
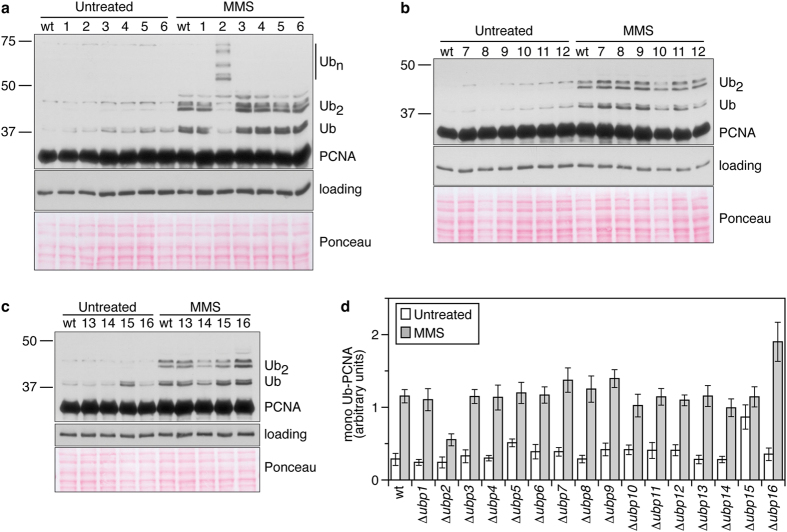
ubPCNA accumulation in fission yeast strains defective in specific USP-family ubiquitin proteases. (**a**–**c**) Ubiquitylated PCNA accumulation in exponentially growing (untreated) or methyl methanesulfonate (MMS)-treated single *ubp1* to *ubp16* deletions in *S. pombe*. Wild-type and single mutant cells exponentially grown at 32 °C were treated for 3 hours with 0.01% MMS. Western blotting was used to analyse TCA-cell extracts for PCNA ubiquitylation, which was quantified and plotted. Cell extracts were resolved in 12% polyacrylamide gels and immunoblotted with affinity purified rabbit α -PCNA antibodies or α -tubulin (loading). Single *ubp1* to *ubp16* deletions in *S. pombe* are indicated by numbers. (**d**) Graph of mono-ubiquitylated PCNA accumulation in untreated and 0.01% MMS-treated single *ubp1–16* deletions *in S. pombe*. Average values of the relative levels of mono-ubiquitylated PCNA from two independent assays are plotted. Error bars were calculated from two independent experiments and indicate the standard deviation.

**Figure 2:**
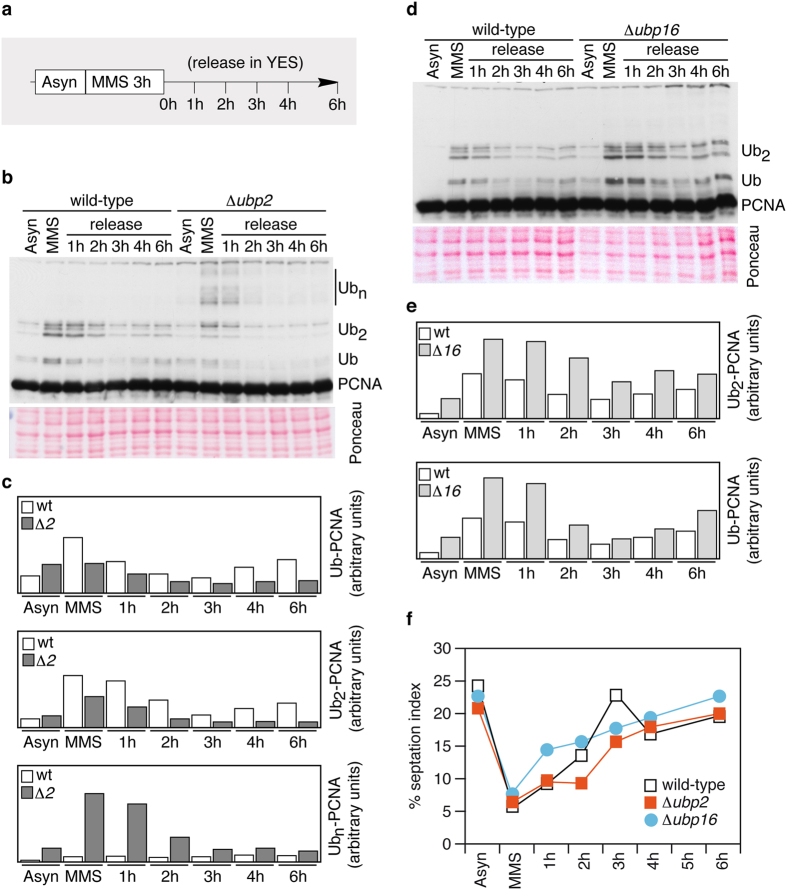
Fission yeast cells lacking *ubp2*^+^ or *ubp16*^+^ deubiquitylate ubPCNA after MMS-induced DNA damage. (**a**) Experimental design; exponentially growing cultures of wild-type, Δ *ubp2*, and Δ *ubp16* strains were treated with 0.02% MMS and then released. Samples were taken at indicated intervals for cell cycle and Western analysis. (**b**) Western blot analysis in wild-type and Δ *ubp2* mutant cells. ubPCNA signals were quantified and normalized to loading controls. (**c**) Quantification is shown in bar diagrams. (**d**) Western blot analysis in wild-type and Δ *ubp16* mutant cells. As was previously performed, ubPCNA signals were quantified and normalized to loading controls. (**e**) Quantification is shown in bar diagrams. (**f**) Plots of septated (septation index) cells of the indicated strains in (**b**,**d**) are shown.

We reasoned that in fission yeast, the ubiquitylation of PCNA might be a reversible process catalysed by deubiquitylating enzymes (or DUBs), as is the case in budding yeast. We have previously shown that that the histone H2B^K123^ ubiquitin protease Ubp10 deubiquitylates ubPCNA in *S. cerevisiae*^11^. Budding yeast Ubp10 is a nucleolar ubiquitin protease of the USP family that is orthologous to Ubp16 in the distantly related fission yeast *S. pombe*^19^. Regarding PCNA modification, fission yeast is an interesting model organism because the sliding clamp is ubiquitylated during each S-phase even in the absence of exogenous DNA damage^20^. This is a strong difference from the budding yeast model in which ubPCNA is poorly detectable in undamaged cells^11^,^21^,^22^. The periodicity of PCNA ubiquitylation throughout the cell cycle (and our own observations) suggests the existence of an active deubiquitylating mechanism during S-phase^20^. Prompted by our interest in studying the potential conservation of the role of the USP family of ubiquitin proteases in PCNA deubiquitylation, we first analysed single mutants of every USP-subfamily DUB of *S. pombe,* looking for defects resulting in the accumulation of ubiquitylated forms of PCNA (Fig. 1). The fission yeast genome encodes 14 putative DUBs that belong to the USP subfamily (from *ubp1*^+^ to *ubp16*^+^). There are two other genes (*ubp10*^+^ and *ubp13*^+^) that encode proteins with DUB domains; however, they lack the full catalytic region required for protease activity^19^. To detect modified forms of the PCNA clamp, we used a previously characterized polyclonal antibody^20^ that specifically detects endogenous PCNA in *S. pombe* (TCA) cell extracts (Fig. S1). As shown in Fig. 1, Δ *ubp2*, Δ *ubp15*, and Δ *ubp16* single mutants showed different defects. Δ *ubp2* mutant cells accumulated PCNA forms with long ubiquitin chains (Fig. 1a, Fig. S2), Δ *ubp15* mutants accumulated mono-ubiquitylated PCNA (plotted in Fig. 1d), particularly in asynchronous cultures (without exogenous DNA damage), and Δ *ubp16* defective cells accumulated mono- and di-ubiquitylated PCNA (Fig. 1c). We then studied the ability of single Δ *ubp2* and Δ *ubp16* mutants to deubiquitylate ubPCNA upon release from HU or MMS treatments and found that, despite the fact that Δ *ubp2* and Δ *ubp16* single mutants accumulated more ubPCNA than wild-type controls, these single mutant cells efficiently deubiquitylated ubPCNA as observed in time-course assays (Fig. 2).

We next overexpressed every USP subfamily member controlled by the extensively used *nmt1*^+^ promoter of fission yeast and found that *ubp12*^+^ and *ubp16*^+^ efficiently reversed PCNA ubiquitylation, whereas *ubp15*^+^ overexpression partially impaired PCNA ubiquitylation (Fig. S3). Unexpectedly, *ubp2*^+^ overexpression did not affect the ubiquitylation of the sliding clamp. Strikingly, *ubp12*^+^ overexpression caused the deubiquitylation of ubPCNA, despite the fact that Δ *ubp12* mutants have no observable defects in ubPCNA accumulation (Fig. 1C). We reasoned that this might be due to the redundant role of another USP subfamily (DUB family) member and initially concentrated our analysis on *ubp12*^+^, *ubp15*^+^, and *ubp16*^+^.

Analysis of the double mutants Δ ubp12 Δ ubp15 and Δ ubp12 Δ ubp16 and the triple mutant *Δ*ubp12 Δ ubp15 Δ ubp16 suggested a role for *ubp12*^+^ in ubPCNA deubiquitylation. Notably, the Δ ubp12 Δ ubp15 Δ ubp16 mutant accumulated high levels of ubPCNA and had an extended S-phase cell cycle (as elutriation assays revealed, Fig. S4). However, this Δ *ubp12* Δ *ubp15* Δ *ubp16* triple mutant showed a poor growth phenotype that made it difficult to draw firm conclusions regarding the cell cycle analysis of PCNA deubiquitylation without further study (Fig. S4). Fully aware that *ubp12*^+^, *ubp15*^+^, and *ubp16*^+^ have additional roles in other cellular processes^19^ (and our own observations) and to minimize the mutation impact, we constructed a triple mutant in which both *ubp12*^+^ and *ubp15*^+^ were fused to a nuclear exclusion signal (NES) that efficiently prevented their accumulation in the nucleus and mimicked the defects in ubPCNA accumulation observed in single, double, or triple mutants (as shown for Ubp15 in Fig. S5). As expected, this *ubp12-NES ubp15-NES* Δ *ubp16* double-NES single deletion mutant grew better than the triple deletant and only showed a mild growth phenotype (further described later). Consistent with a role in the deubiquitylation of the sliding clamp, *ubp12-NES ubp15-NES* Δ *ubp16* cells accumulated high levels of ubPCNA (as shown later). Furthermore, the analysis of PCNA in this triple mutant in undamaged cells or cells blocked early in S-phase with hydroxyurea (HU) or cells treated with the alkylating agent methyl methanesulfonate (MMS) indicated that these three ubiquitin proteases were major players in reverting PCNA ubiquitylation during S-phase in fission yeast.

### Multiple UBPs with specific roles, at specific sub-nuclear locations, are involved in ubPCNA deubiquitylation in the fission yeast *S. pombe*

The subcellular localization of *S. pombe* DUBs has been studied in depth^19^. Consistent with these data, we observed that Ubp16 localizes to the nucleolus (Fig. S5), whereas Ubp15 is present in the nucleus and in unidentified cytoplasmic spots/structures (Fig. S5) but does not co-localize with Ubp16 in the nucleolus (Fig. S5). GFP-tagged Ubp12 was also detected in the nucleus, and these data are consistent with the hypothesis that Ubp16 deubiquitylates PCNA in the nucleolus, whereas Ubp15 and Ubp12 primarily deubiquitylate non-nucleolar ubPCNA elsewhere in the nucleus. The combined analysis of nucleolar Ubp16 (Δ *ubp16*^424–457^-GFP) and nuclear Ubp12 and Ubp15 (*ubp12-NES* and *ubp15-NES*) mutants confirmed this hypothesis.

**Figure 3:**
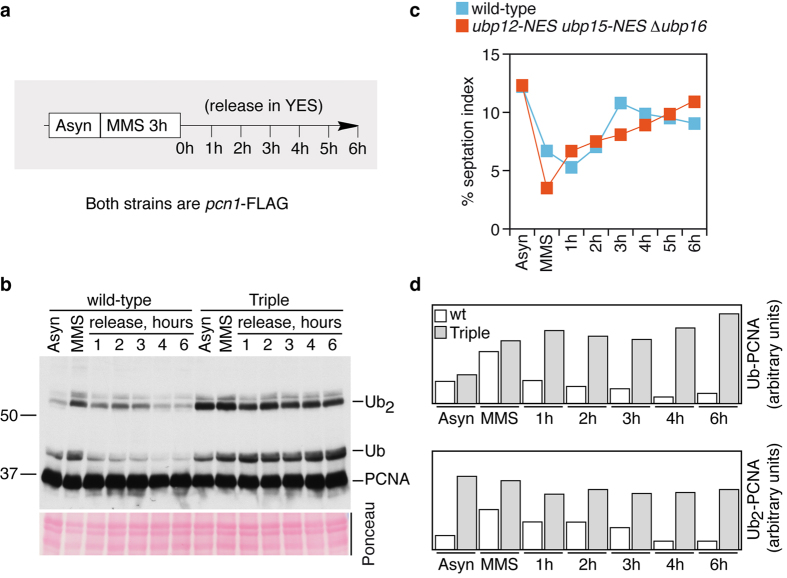
Ubp12, Ubp15, and Ubp16 are required for ubPCNA deubiquitylation after MMS-induced DNA damage. (**a**) Experimental design; exponentially growing cultures of wild-type and *ubp12-NES ubp15-NES* Δ *ubp16* strains were treated for 3 hours with 0.01% MMS, washed twice in fresh pre-warmed media, and then released in YES media (in the absence of the alkylating chemical). Samples were taken at indicated intervals for cell cycle and Western analysis. Note that both strains were *pcn1*-FLAG tagged to detect PCNA. (**b**) Immunodetection of PCNA and modified PCNA forms with α -FLAG. (**c**) Percentage of septated cells (septation index) during the time-course experiment. (**d**) Mono- and di-ubiquitylated PCNA forms were quantified, normalized, and plotted.

We next analysed the reversal of PCNA ubiquitylation in control and *ubp12-NES ubp15-NES* Δ *ubp16* strains upon release from MMS-mediated DNA damage, and deubiquitylation was blocked in the triple mutant (Fig. 3). This suggested that Ubp12, Ubp15, and Upb16 are all important to revert PCNA ubiquitylation throughout S-phase.

**Figure 4:**
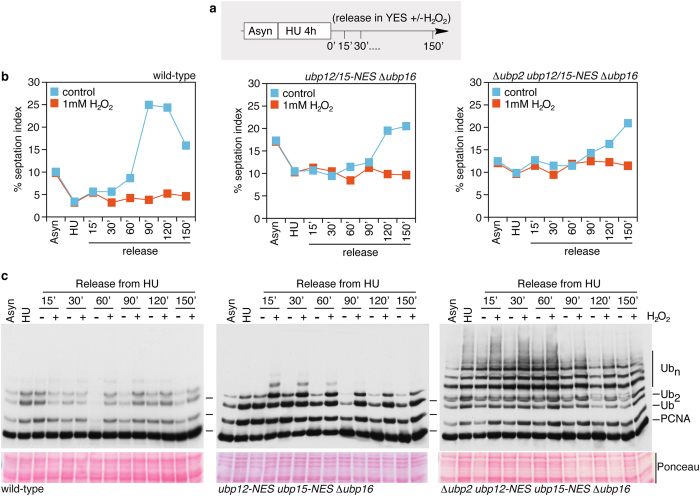
Analysis of PCNA ubiquitylation and cell cycle progression in *S. pombe* mutant cells defective in *ubp2*^+^, *ubp12*^+^, *ubp15*^+^, and *ubp16*^+^. (**a**) Experimental design; exponentially growing cultures of wild-type, *ubp12-NES ubp15-NES* Δ *ubp16*, and Δ *ubp2 ubp12-NES ubp15-NES* Δ *ubp16* strains (as indicated) were synchronized early in S-phase by treating them for 4 hours with 20 mM HU, washed twice in fresh pre-warmed media, and then released in YES media in the absence (− ) or in the presence (+ ) of 1 mM H_2_O_2_. Samples were taken at indicated intervals for cell cycle and Western analysis. (**b**) Percentage of septated cells (septation index) during the time-course experiment in the indicated strains. (**c**) Immunodetection of PCNA and modified PCNA forms with α -FLAG. Note that the three strains were *pcn1*-FLAG tagged to detect PCNA.

Despite high levels of accumulated ubiquitylated PCNA (Fig. S6), we also found that triple mutant *ubp12-NES ubp15-NES* Δ *ubp16* cells deubiquitylate ubPCNA upon release from HU (Fig. 4, middle graph and Western blot), indicating that at least one additional DUB is involved in the deubiquitylation of the sliding clamp. Significantly, we also noted that *ubp12-NES ubp15-NES* Δ *ubp16* triple mutant cells show a cell cycle delay; they progress slowly from the HU block in early S-phase to cytokinesis.

### The unique role of Ubp2 in ubPCNA deubiquitylation

We observed that wild-type cells accumulate PCNA with long ubiquitin chains when exposed to hydrogen peroxide (closely resembling *ubp2* mutants). K63-linked polyubiquitylation has been associated with the inactivation of *S. cerevisiae* Ubp2 in response to oxidative stress^23^. Some DUBs can be reversibly inactivated through the oxidation of their catalytic cysteine by H_2_O_2_^24^, and fission yeast *ubp2* mutants accumulate K63-linked polyubiquitylated PCNA (Figs 1 and 2). Thus, we predicted that Ubp2 would revert PCNA ubiquitylation in a concerted manner with Ubp12, Ubp15, and Ubp16 to fully deubiquitylate the sliding clamp (particularly in response to endogenous and exogenous oxidative DNA damage). We reasoned that the periodic pattern of PCNA ubiquitylation and deubiquitylation would be lost in cells lacking all relevant fission yeast PCNA-DUBs. Therefore, we next combined Δ *ubp2* with *ubp12-NES ubp15-NES* Δ *ubp16* and evaluated ubPCNA stability in time-course experiments with HU-presynchronized cells released in the absence or in the presence of hydrogen peroxide. Upon release from the HU arrest in early S-phase, ubiquitylated PCNA remained fairly stable over time in quadruple mutant cells, which was similar to cells fully defective in PCNA deubiquitylation and released in hydrogen peroxide (Fig. 4). We also found that Δ *ubp2 ubp12-NES ubp15-NES* Δ *ubp16* quadruple mutant cells have a strong cell cycle delay. Together, these converging lines of evidence suggest that Ubp2, Ubp12, Ubp15, and Ubp16 are major (K164) ubPCNA ubiquitin proteases in fission yeast.

As shown above, we also noticed the accumulation of PCNA with long ubiquitin chains in *∆ubp2* cells treated with MMS (Figs 1 and 2), but these chains were removed efficiently when the DNA damage was removed. To test whether the removal of these long chains on PCNA was dependent upon Ubp12/15/16, we studied Δ *ubp2 ubp12-NES ubp15-NES* Δ *ubp16* cells. Upon release from DNA damaging conditions, PCNA deubiquitylation was blocked in the quadruple mutant. Although Ubp2 is particularly important for the removal of long ubiquitin chains from PCNA in the presence of DNA damage, it appears that Ubp12/15/16 can cooperate to remove these chains from PCNA once the damage has been removed.

Next, we tested the importance of PCNA deubiquitylation *in vivo*; we reasoned that impeding a relevant S-phase event should have an impact on cell-cycle progression. We found that the combination of Δ *ubp2*, *ubp12-NES*, *ubp15-NES*, and Δ *ubp16* leads to growth defects and increased sensitivity to replication stress induced by HU treatment or DNA damage (Fig. S7).

### Ubp2 deconjugates K63-linked poly-ubiquitin chains from K164 poly-ubiquitylated PCNA *in vitro*

**Figure 5:**
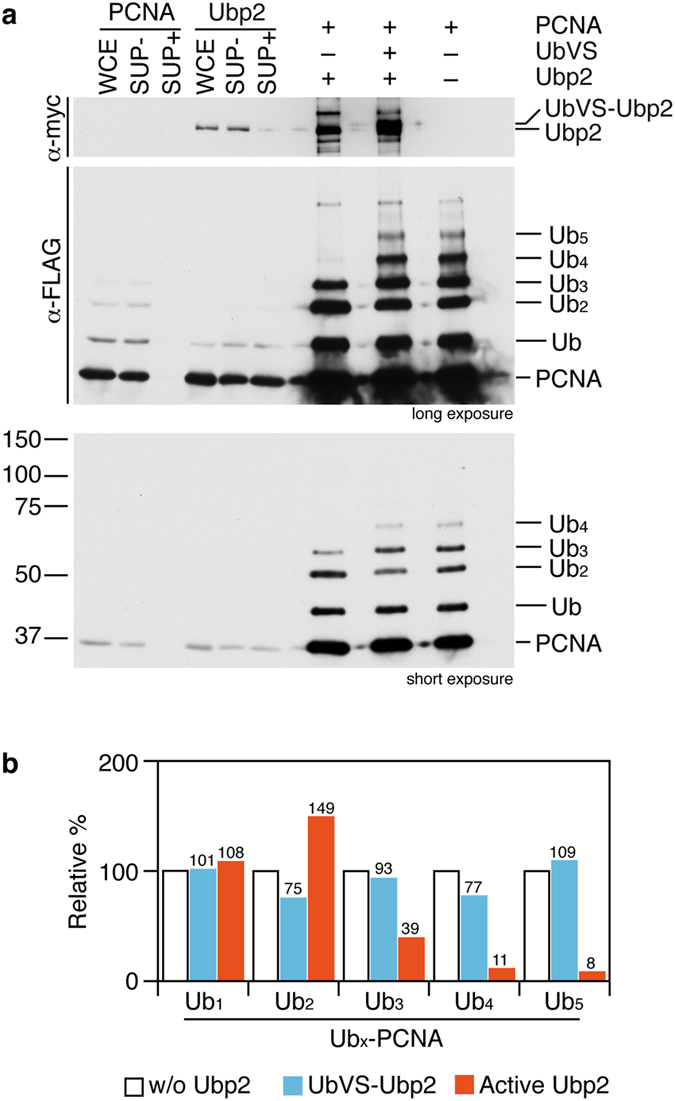
Ubp2 removes long K63-linked ubiquitin chains from poly-ubiquitylated PCNA *in vitro*. (**a**) Ubp2-myc deubiquitylates *in vitro* polyubiquitylated PCNA. Mono-, di-, and poly-ubiquitylated (ub_3_-to-ub_6_) PCNA was obtained by immunoprecipitation with anti-FLAG antibody from a Δ *ubp2 ubp12-NES ubp15-NES* Δ *ubp16 pcn1-FLAG* strain (synchronized in early S-phase; 3 hours 20 mM HU). Immunoprecipitated samples were divided in three; two of the aliquots were incubated with immunoprecipitated Ubp2-myc in the absence or in the presence of UbVS to inhibit the protease activity of Ubp2 (as described in the Methods section). The third aliquot served as a reference sample of the immunoprecipitated PCNA. PCNA deubiquitylation was detected by the distinctive SDS-PAGE gel mobility of the different PCNA forms. Whole cell extracts (WCE), depleted supernatants (SUP+ ), and non-depleted supernatants (SUP− ) from PCNA-FLAG and Ubp2-myc strains are also shown. (**b**) Signals of the different PCNA forms in the deubiquitylation reactions and controls resolved in the Western blots were quantified and plotted.

We next tested the ability of immunoprecipitated Ubp2-myc protein to deubiquitylate PCNA in an *in vitro* (ubiquitin protease) assay using mono- and poly-ubiquitylated PCNA-FLAG (ub-, ub_2_-, ub_3_-, ub_4_- and ub_5_-PCNA-FLAG) as a substrate immunoprecipitated from a Δ *ubp2 ubp12-NES ubp15-NES* Δ *ubp16 pcn1*-FLAG strain (Fig. 5). Ubp2-myc efficiently deubiquitylated ub_5_-PCNA, ub_4_-PCNA, and, less efficiently, Ub_3_-PCNA, but it was unable to deubiquitylate ub- or ub_2_-PCNA. In fact, ub_2_-PCNA forms accumulated in the *in vitro* reactions, suggesting that polyubiquitylated PCNA with 3 or more ubiquitin moieties was converted into di-ubiquitylated PCNA by Ubp2. This *in vitro* ubiquitin protease activity of Ubp2 towards polyubiquitylated PCNA was directly inhibited by binding UbVS to the enzyme prior to the protease reaction, indicating that an active Ubp2-associated protease activity is required to remove these unusually long polyubiquitin chains from PCNA. These experiments support the hypothesis that Ubp2 deubiquitylates K63-linked ub_3_-to-ub_5_-polyubiquitylated PCNA forms.

### Ubp15 and Ubp16 deconjugate ubiquitin and K63-linked di-ubiquitin chains from K164 mono- and di-ubiquitylated PCNA *in vitro*

**Figure 6:**
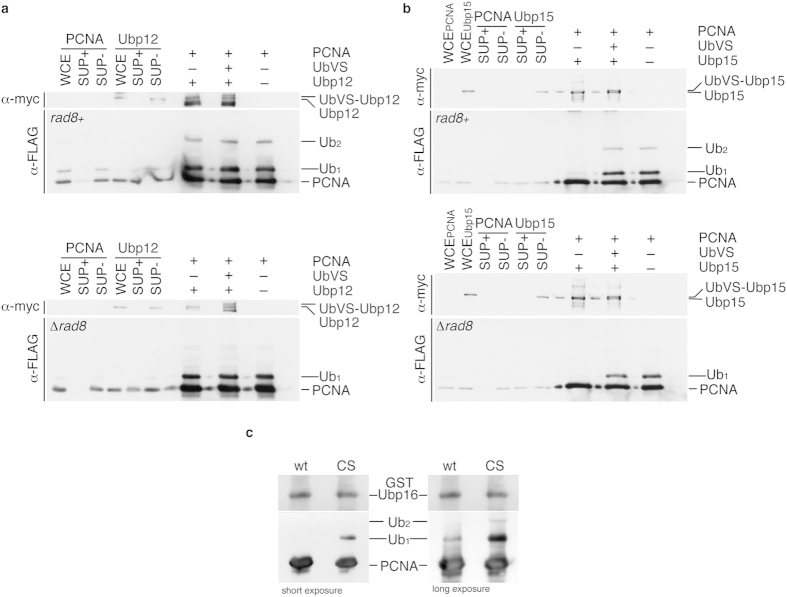
Ubp15 and Ubp16 deubiquitylate mono- and di-ubiquitylated PCNA *in vitro*. Mono- and di-ubiquitylated PCNA was obtained by immunoprecipitation with anti-FLAG antibody from a *ubp12-NES ubp15-NES* Δ *ubp16 pcn1-FLAG* strain (*rad8*^+^ blot) and mono-ubiquitylated PCNA was obtained from a *ubp12-NES ubp15-NES* Δ *ubp16* Δ *rad8 pcn1-FLAG* strain (Δ *rad8* blot) (both strains were synchronized in early S-phase; 3 hours 20 mM HU). In each case (*rad8*^+^ and Δ *rad8*), immunoprecipitated samples were divided in three, and two of the aliquots were incubated with immunoprecipitated Ubp12-myc or Ubp15-myc (as indicated) in the absence or in the presence of UbVS to inhibit any UBP activity (as described in the Methods section). The third aliquot served as a reference sample of the immunoprecipitated PCNA. PCNA deubiquitylation was detected by the distinctive SDS-PAGE gel mobility of the different PCNA forms. Whole cell extracts (WCE), depleted supernatants (SUP+ ), and non-depleted supernatants (SUP− ) from PCNA-FLAG and Ubp12-myc or Ubp15-myc strains are also shown. (**a**) Active Ubp12 fails to deubiquitylate PCNA *in vitro*. (**b**) Immunoprecipitated Ubp15-myc deubiquitylates both mono- (ub_1_) and di-ubiquitylated (ub_2_) PCNA *in vitro*. (**c**) GST-Ubp16 deubiquitylates ub_1_-PCNA and ub_2_-PCNA *in vitro*. Purified GST-Ubp16 and GST-Ubp16^CS^ proteins from *E. coli* were assayed *in vitro* for their ability to deubiquitylate PCNA. Note that GST-Ubp16 but not the catalytically inactive form GST-Ubp16^CS^ (C134S) deconjugated ubiquitin from mono- and di-ubiquitylated PCNA.

Having shown *in vitro* that Ubp2 removes ubiquitin and ubiquitin chains from polyubiquitylated PCNA (with 3 or more ubiquitin moieties), thus indicating that Ubp2 is an (ub_3_-to-ub_8_PCNA) endo-deubiquitylase, we reasoned that perhaps the Ubp2 function *in vivo* is to shorten polyubiquitylated PCNA (›ub_3_PCNA) to allow Ubp12, Ubp15, and Ubp16 to fully deubiquitylate the sliding clamp. If this hypothesis is correct, we should be able to assemble an *in vitro* reaction and show that Ubp12, Ubp15, and Ubp16 remove ubiquitin from mono- and di-ubiquitylated PCNA. Therefore, we tested the activity of Ubp12, Ubp15, and Ubp16 in ubiquitin protease assays using mono- and di-ubiquitylated PCNA as substrates immunoprecipitated from *ubp12-NES ubp15-NES* Δ *ubp16 pcn1*-FLAG (as a source of ub_1_- and ub_2_-PCNA) and *ubp12-NES ubp15-NES* Δ *ubp16* Δ *rad8 pcn1*-FLAG (as a source of ub_1_-PCNA) strains (Fig. 6). In our *in vitro* enzymatic assays, Ubp12 failed to deubiquitylate ub_1_- or ub_2_-PCNA. However, we found that Ubp15-myc, Ubp16-myc, and GST-Ubp16 efficiently deubiquitylated both mono- and di-ubiquitylated PCNA forms (Fig. 6B,C, Fig. S8). These experiments, together with *in vivo* evidence, support the conclusion that Ubp15 and Ubp16 are substrate/PCNA-specific DUBs that deubiquitylate K164 mono-ubiquitylated PCNA and (K63-linked) K164 di-ubiquitylated PCNA.

### Mutation of Rhp18^Rad18^ or Rad8^Rad5^ PCNA-ubiquitin protein ligases suppresses PCNA-DUBs-associated cell cycle phenotypes

**Figure 7:**
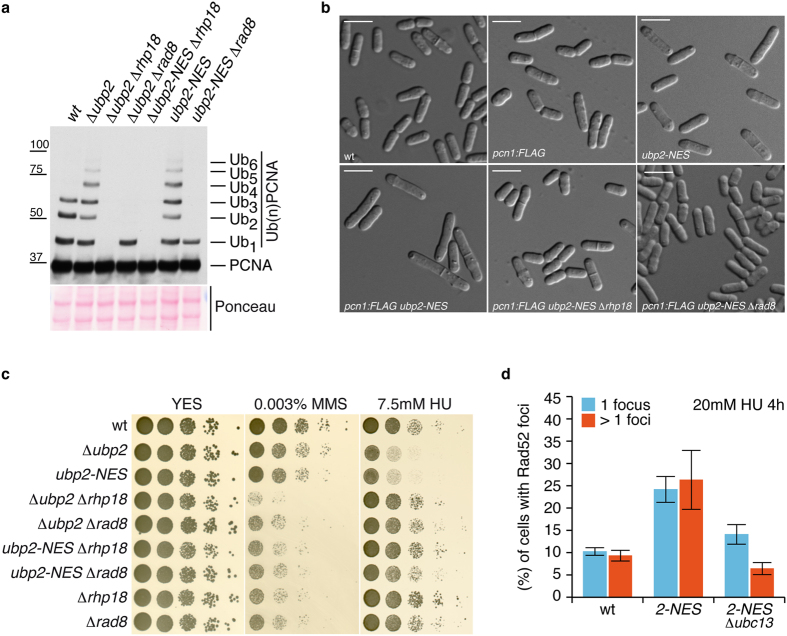
*ubp2-NES*-associated cell elongation phenotype is suppressed by preventing K164 PCNA ubiquitylation. (**a**) Immunodetection of ubiquitylated forms of PCNA-FLAG in *pcn1*-FLAG (wt), *pcn1*-FLAG Δ *ubp2*, *pcn1*-FLAG Δ*ubp2* Δ *rhp18*, *pcn1*-FLAG Δ *ubp2 Δrad8*, *pcn1*-FLAG *ubp2-NES*, *pcn1*-FLAG *ubp2-NES* Δ *rhp18*, and *pcn1*-FLAG *ubp2-NES* Δ *rad8* cell extracts to show that the accumulation of (long K63-linked ubiquitin chained) polyubiquitylated PCNA forms in *ubp2* mutant cells depends on the K164 monoubiquitylation of PCNA (Rhp18) and the K63-linked di-ubiquitylation of mono-ubiquitylated PCNA (Rad8). (**b**) Optical Nomarski images of wild-type, *pcn1*-FLAG, *pcn1*-FLAG *ubp2-NES*, *pcn1*-FLAG *ubp2-NES Δrhp18*, and *pcn1*-FLAG *ubp2-NES* Δ *rad8* cells (as indicated). Note that the elongated cell phenotype in *pcn1*-FLAG *ubp2-NES* cells is suppressed by Δ*rhp18* or Δ*rad8* deletion. (**c**) Epistasis analysis of Δ *rhp18*, Δ*rad8*, and *ubp2* mutant alleles. Ten-fold dilutions of the indicated strains incubated at 32 °C on YES plates with or without MMS or HU (as indicated). Note that Δ*ubp2* Δ *rhp18*, Δ *ubp2* Δ *rad8*, *ubp2-NES* Δ *rhp18*, and *ubp2-NES* Δ *rad8* double mutants behave like single Δ *rhp18* or Δ *rad8* mutants, indicating that Δ *rhp18* and Δ *rad8* are epistatic to Δ *ubp2* and *ubp2-NES*. (**d**) The *ubp2-NES* allele accumulates Rad52-foci in a Ubc13-dependent manner. (wt) wild-type, (*2-NES*), *ubp2-NES* and (*2-NES* Δ *ubc13*), *ubp2-NES* Δ *ubc13.* Bar graph of nuclei containing single or multiple Rad52-foci in fission yeast strains after 4 hours of 20 mM HU treatment. The results are representative of three independent experiments to obtain an estimate of error (where n is greater than 1,200 for each sample).

**Figure 8:**
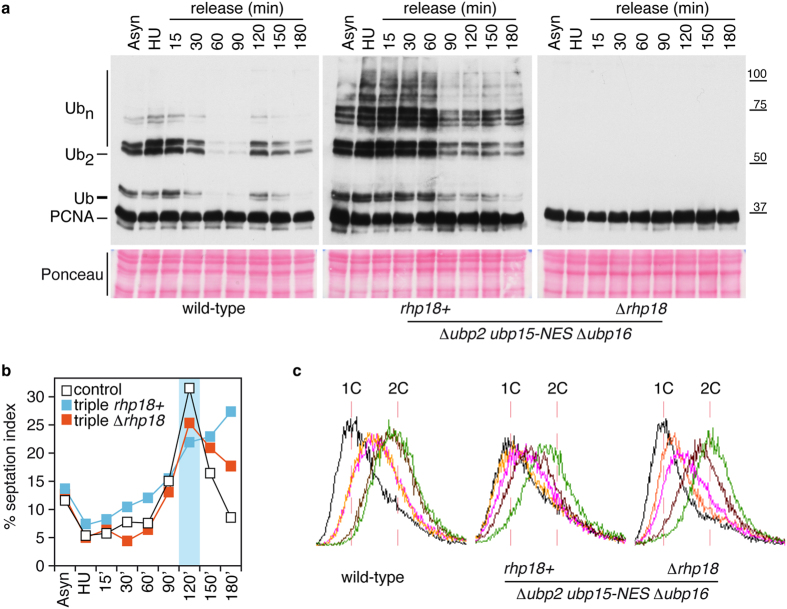
Rescue of cell cycle phenotypes in Δ*ubp2 ubp15-NES* Δ*ubp16* triple mutants through the suppression of K164-linked PCNA ubiquitylation. Experimental design; exponentially growing cultures of wild-type, Δ *ubp2 ubp15-NES* Δ *ubp16* (triple), and Δ *ubp2 ubp15-NES Δubp16* Δ *rhp18* strains (as indicated) were synchronized early in S-phase by treating them for 4 hours with 20 mM HU, washed twice in fresh pre-warmed YES media, and then released. Samples were taken at indicated intervals for cell cycle position and Western analysis. (**a**) Immunodetection of ubiquitylated forms of PCNA-FLAG in *pcn1*-FLAG (wt), *pcn1*-FLAG Δ *ubp2 ubp15-NES* Δ *ubp16*, and *pcn1*-FLAG Δ *ubp2 ubp15-NES* Δ *ubp16* Δ *rhp18* cell extracts to show that the accumulation of (long K63-linked ubiquitin chains) polyubiquitylated PCNA forms in these triple mutant cells depends on the K164 monoubiquitylation of PCNA (Rhp18). (**b**) Percentage of septated cells (septation index) during the time-course experiment in the indicated strains. (**c**) DNA content analysis by FACS of cell samples synchronized in HU (black lines) and 15 (orange lines), 30 (pink lines), 60 (brown lines), and 90 (green lines) minutes after the release in YES. Note that the S-phase and septation delays of Δ *ubp2 ubp15-NES* Δ *ubp16* triple mutants were reverted in a Δ *rhp18* background.

As previously shown, *ubp12-NES ubp15-NES* Δ *ubp16* triple mutant cells show a solid cell cycle delay phenotype when combined with Δ *ubp2* (graphs in Fig. 4). This additive effect suggested that *ubp2* mutants have a mild cell cycle phenotype that is easier to detect in combination with mutations of other ubiquitin proteases controlling PCNA deubiquitylation. In the course of our studies, we found that *ubp2* mutant cells in combination with *pcn1*-FLAG were elongated compared to the wild-type or *pcn1*-FLAG controls (Fig. 7). It is important to mention that neither *pcn1-FLAG* cells nor *ubp2* mutants have a strong phenotype on their own (Fig. 7). We reasoned that if there is a link between this elongated cell phenotype and the accumulation of ubiquitylated PCNA, preventing PCNA ubiquitylation would rescue this cell cycle defect. Therefore, we examined the *pcn1-FLAG ubp2-NES*/Δ *ubp2*-associated cell cycle phenotype in Δ *rhp18* or Δ *rad8* mutant strains defective in the E3 ubiquitin protein ligases that control PCNA mono- and poly-ubiquitylation, respectively^20^. Significantly, the cdc-like phenotype of *ubp2-NES* and Δ *ubp2* was efficiently suppressed by preventing K164 PCNA ubiquitylation (in a Δ *rhp18* mutant) or polyubiquitylation (in a Δ *rad8* mutant) because the cell size of exponentially growing cells from *ubp2-NES* Δ *rhp18*, Δ *ubp2* Δ *rhp18, ubp2-NES* Δ *rad8,* or Δ *ubp2* Δ *rad8* mutants was indistinguishable from the cell size of wild-type controls. These results suggest that Ubp2 has a direct role in PCNA deubiquitylation that is relevant for normal cell cycle progression in fission yeast. We also tested whether the deletion of *rhp18*^+^ would suppress the slow S-phase phenotype of the Δ *ubp2 ubp12-NES ubp15-NES* Δ *ubp16* quadruple mutant (Fig. 4), but this quintuple mutant grew poorly and prevented analysis requiring presynchronization. However, further support for the cell cycle role of PCNA deubiquitylation comes from evidence regarding the rescue of the S-phase and septation delays of a Δ *ubp2 ubp15-NES* Δ *ubp16* triple mutant by deleting the PCNA ubiquitin ligase Rhp18 (Fig. 8). All the evidence suggests that PCNA deubiquitylation is an important cell cycle event in *S. pombe*.

### S-phase Rad52-dependent template switching analysis in fission yeast strains with specific defects in ubPCNA deubiquitylation

In this work, we have related the deubiquitylation of PCNA to the enzymatic activity of the USP-family DUBs Ubp2, Ubp12, Ubp15, and Ubp16 in the nucleus. Notably, we observed that *ubp2*, *ubp15*, and *ubp16* single mutants show specific defects regarding ubPCNA accumulation *in vivo* (Figs 1 and 2, Fig. S2). On the basis of these results, we were interested in understanding whether these unique defects are associated with specific physiologically relevant phenotypes, particularly those linked to tolerance to DNA damage during S-phase. In budding and fission yeast, Rad52 has been associated with the template switching branch of the tolerance to DNA damage pathway^25^,^26^,^27^,^28^; for this reason, we studied the accumulation of Rad52 in nuclear foci in *ubp2*, *ubp12*, *ubp15*, and *ubp16* mutant cells blocked/synchronized in early S-phase with HU. We determined the number and percentage of cells accumulating single or multiple nuclear Rad52 foci after a 4-hour block in HU (Fig. 7D, Fig. S9). Consistent with a lower number of template switching events, we observed a decrease in the percentage of cells with nuclear Rad52 foci in *ubp15* and *ubp16* mutant cells in early S-phase compared to controls (Fig. S9). Interestingly, we found that *ubp2* mutant cells accumulate single and multiple Rad52 foci. We also found that this phenotype depended entirely on PCNA polyubiquitylation because it was efficiently suppressed by the deletion of *ubc13*^+^. Ubc13 is a PCNA E2 K63-polyubiquitin-conjugating enzyme that forms part of the Rad8/Mms2-Ubc13 complex that controls translesion synthesis in *S. pombe*^29^. This evidence suggests that in fission yeast the accumulation of long polyubiquitylated PCNA forms enhances Rad52-dependent template switching in early S-phase.

## Discussion

In this study, we explain how ubPCNA is deubiquitylated in *S. pombe* due to the concerted nuclear activity of four different members of the USP family of ubiquitin proteases: Ubp2, Ubp12, Ubp15, and Ubp16. Defects in Ubp15 primarily result in the accumulation of mono-ubPCNA, particularly during the S-phase of a normal cell cycle. However, this *∆ubp15* does not affect PCNA ubiquitylation upon the treatment of cells with DNA damaging agents. In contrast, Ubp16 appears to deubiquitylate mono- and di-ubPCNA in the nucleolus in response to DNA damage, whereas Ubp12 has the same activity in the non-nucleolar fraction of the nucleus. Ubp12 might act redundantly with Ubp15 because the mutation of *ubp12*^+^ lacks a phenotype but greatly enhances the phenotype of *ubp15* single and *ubp15 ubp16* double mutants. The fourth DUB to regulate PCNA, Ubp2, removes long K63-linked ubiquitin chains that accumulate in wild-type cells in response to DNA damage. All four ubiquitin proteases are active throughout the cell cycle and likely deubiquitylate the sliding clamp in response to the presence of ubPCNA during S-phase. Significantly, their concerted role in reverting PCNA ubiquitylation is important for cell cycle progression and, particularly, for cells to respond to DNA damage or replication blocks. A multiple/quadruple mutant (Δ *ubp2 ubp12-NES ubp15-NES* Δ *ubp16*), which is fully defective in ubPCNA deubiquitylation, is viable, but the cells appeared elongated. This cdc-like phenotype depended at least in part on the *ubp2* mutation because it was reverted/suppressed by preventing PCNA ubiquitylation and/or polyubiquitylation, indicating that the cell cycle phenotype is the consequence of the excess PCNA ubiquitylation. Further support for this hypothesis comes from the observation that links PCNA ubiquitylation with the S-phase delay detected in Δ *ubp2 ubp15-NES* Δ *ubp16* triple mutants.

Our observations are consistent with the idea that the impairment of ubPCNA deubiquitylation leads to an extended S-phase in *S. pombe*, and they suggest that the timely removal of PCNA ubiquitylation after lesion bypass is necessary to ensure processive DNA replication. In eukaryotes, lesion bypass at replication forks relies on polymerase switching, a replicative-to-TLS DNA polymerase exchange based on PCNA ubiquitylation^16^,^30^,^31^,^32^,^33^. Recent studies suggest that PCNA deubiquitylation is required after lesion bypass synthesis to resume normal DNA synthesis by Polδ 12,34. In particular, it has been shown that PCNA monoubiquitylation prevents Polδ from replacing Polη in a budding yeast *in vitro* reconstituted system^34^. In human cell lines, the replacement of Polη by replicative DNA polymerases (in TLS termination) is more complex; three alternative mechanisms have been described, including DVC1/SPARTAN accumulation at stalled forks^35^, PAF15 binding to PCNA^36^, and USP10-dependent ISGylated-PCNA deubiquitylation^12^. However, a key unanswered question is whether PCNA deubiquitylation is an S-phase-specific modification, thus allowing a rapid resumption of processive DNA replication after lesion bypass. Here, we provide the first *in vivo* evidence that links the deubiquitylation of the sliding clamp with cell cycle progression control during S-phase.

Tolerance to DNA damage is based on a non-processive DNA replication event called translesion synthesis, which is either a low-fidelity mechanism (associated with TLS-DNA polymerases) or a complex molecular tool (involving template switching) that can be used to bypass DNA lesions at the replication fork during S-phase^37^,^38^,^39^,^40^. If we hypothesized that ubPCNA deubiquitylation is important to prevent the expanded use of translesion synthesis, we can predict that defects in this deubiquitylating process would eventually lead to an extended S-phase due to the nature of this DNA replication event that will likely slow down DNA synthesis at the replication fork. Furthermore, even if translesion synthesis works primarily during late DNA replication (i.e., S/G2 boundary) defects in ubPCNA deubiquitylation can cause a delay in the completion of S-phase. Therefore, in eukaryotes, translesion synthesis may result in slow DNA replication, as has been shown for DNA polymerase switching in *E. coli*^41^. Our results show that there is a strong correlation between the accumulation of ubiquitylated PCNA and extended S-phase duration.

Our observations force us to ask why the deubiquitylation of ubPCNA in the fission yeast is this complex. It is noteworthy to mention that all PCNA ubiquitin proteases described in this work have other substrates distinct from PCNA^19^. Indeed, we show here that the reversion of PCNA ubiquitylation involves three or likely four different ubiquitin proteases in *S. pombe*. This observation suggests that fission yeast evolved a redundant system to ensure backup activity to maintain the controlled ubiquitylation of the sliding clamp. However, this possibility contradicts the idea that any backup system by gene redundancy is evolutionarily unstable^42^. In fact, we found that each of these ubiquitin proteases is dedicated to the deubiquitylation of a specific subnuclear fraction or a particular type of ubiquitylated PCNA, which in turn would suggest some degree of functional specialization (for further discussion see model in Fig. S10). Taken together, this evidence suggests that ubPCNA deubiquitylation in *S. pombe* is a case of distributed robustness, or perhaps more precisely, a case of division of labour.

The observed accumulation of mono-ubiquitylated PCNA in *ubp15*^+^ mutants, the increase shown in mono- and di-ubiquitylated PCNA in the Δ *ubp16* strain, the synergistic effect of combining mutations in *ubp15*^+^ and *ubp16*^+^, and the evidence regarding the overexpression of both genes together with the *in vitro* data provide robust evidence that Ubp15 and Ubp16 revert the K164 mono- and di-ubiquitylation of PCNA. By reverting the ubiquitylation of PCNA, Ubp15 and Ubp16 activities counterbalance the ubiquitin ligase activity of the Rhp6/Rhp18 complex responsible for PCNA mono-ubiquitylation. Therefore, these two ubiquitin-specific proteases have the potential to be part of a safeguard mechanism limiting the residence time of TLS DNA polymerases in the replicating chromatin. In this context, an important observation presented in this work is that Ubp15 and Ubp16 can remove both mono- and di-ubiquitin from PCNA *in vivo* and *in vitro*, likely acting as substrate-specific DUBs. We have reported similar *in vivo* findings in budding yeast with Ubp10, a DUB related to fission yeast Ubp16^11^. Therefore, our results clearly suggest that these ubiquitin-specific proteases are crucial to downregulate simultaneously both error-prone and error-free branches of the tolerance pathway in fission yeast. The activity of Ubp15 and Ubp16^Ubp10^ would ensure a switch back to normal processive DNA replication without favouring a particular branch of the tolerance pathway. Finally, Ubp2 activity accumulates di-ubiquitylated PCNA. Therefore, together these three fission yeast ubiquitin proteases prevent an increment in mono-ubiquitylated PCNA that could eventually boost the unwanted interaction between TLS-polymerases and PCNA (error-prone and, thus, potentially mutagenic).

In the budding yeast, recent evidence demonstrated that DNA damage bypass-dependent PCNA ubiquitylation can be uncoupled from genome replication and is functional outside the limits of S-phase^43^,^44^, where it usually takes place^43^,^45^. It is already known that in fission yeast, as in budding yeast, PCNA ubiquitylation normally occurs during S-phase^20^,^43^. Here, our experiments confirm that *S. pombe* cells ubiquitylate and deubiquitylate PCNA during S-phase in a dynamic manner to ensure normal DNA replication, such that the ubiquitylation of PCNA is rapidly followed by the deubiquitylation of the sliding clamp from the beginning to the end of the S phase, likely to limit the window of opportunity for effective translesion synthesis to persist.

## Methods

### Yeast strains and cell culture

All strains used in this study were derived from wild-type 972^h−^. All strains were *leu1-32 ura4-d18* except as noted. Standard molecular biology and genetic methods were used for the manipulation and construction of new strains^46^,^47^. Cultures were grown in appropriately supplemented yeast extract (YES media: yeast extract plus supplements [225 mg/l adenine, histidine, leucine, uracil, and lysine hydrochloride]). *S. pombe* cells were incubated at 30 °C unless otherwise specified. Strains containing repressible *nmt1*-regulated genes or *nmt1*-plasmids were grown in minimal medium (EMM) appropriately supplemented with 225 mg/l adenine and uracil and containing thiamine. Induction was performed by washing exponentially growing cultures twice and resuspending in medium without thiamine as previously reported^48^,^49^. Drug treatments were performed in exponentially cultures grown in YES or EMM that were appropriately supplemented as required (*nmt1*-induction experiments).

### Imaging of Cells

The *in vivo* fluorescence imaging of green fluorescent protein (GFP)-, yellow fluorescent protein (YFP)-, and red fluorescent protein (RFP)-tagged strains and 4′ ,6-diamidino-2-phenylindole (DAPI) staining was performed in a Leica DM 6000B microscope (63X objective; 1,32 Oil Plan-APO) equipped with a Hamamatsu ORCA-ER c4742-95 digital camera and MetaMorph software (Molecular Devices). Fluorescence images were also collected using a Zeiss Axioplan 2 microscope with a 63X or a 100X objective and a digital camera (Hamamatsu ORCA-ER c4742-95) and processed with Openlab 4.0.3 software (Improvision, Coventry, United Kingdom). To quantify Rad52-YFP foci, the appearance at least 1,200 nuclei from three separate experiments was examined for each strain and each time point.

*in vivo* nuclear staining was performed with 4′ ,6-diamidino-2-phenylindole (DAPI) or Hoechst (bisbenzimide H 33342; Sigma, Madrid, Spain) as previously described^49^,^50^. Cells were collected by centrifugation at 3,000 rpm and incubated in DAPI or Hoechst 1× for 15 min. To quantify the percentage of binucleated cells in certain experiments, cells were fixed in 70% ethanol, and then they were washed and resuspended in 1× phosphate-buffered saline (PBS) (8.5 mM Na_2_HPO_4_·12H_2_O, 1.88 mM NaH_2_PO_4_·H_2_O, 130 mM NaCl) containing 1× DAPI as previously described^51^. At least 350 cells were classified as uni- or binucleated in each count. The septation index in wild-type cells and *ubp*2, *ubp*12, *ubp*15, and *ubp*16 mutant cells was determined by counting septated cells *in vivo*.

### Tagging yeast proteins and gene deletion

Gene deletions or modifications were performed by PCR-mediated one-step gene replacement^52^. The selection markers used were KanMX6, HphMX4, or NatMX4, which allow selection with geneticin, hygromycin, and nourseothricin, respectively. We also used the *ura4*^+^ marker. The correct integration in the genome was confirmed by PCR with flanking and internal oligonucleotides and sequencing. In the case of tagged alleles, the expression of tagged proteins was confirmed by Western blotting. For tagging *ubp2*^+^*, ubp12*^+^*, ubp15*^+^*, ubp16*^+^, and *pcn1*^+^ with myc or FLAG, the myc and FLAG sequences were directly introduced at the 3′ end of the ORF of each gene. As indicated, in some experiments we used a tagged *pcn1*^+^ allele (endogenous promoter) to generate a Pcn1-FLAG fusion protein (with the FLAG epitope at the C-terminus). The FLAG-tagged strain behaved similarly to wild-type controls. For tagging with GFP, a flexible glycine-serine linker was introduced between the target protein and the tag.

The nuclear export signal (NES) of the heat-stable inhibitor of cAMP-dependent protein kinase (C-PKI; sequence: LALKLAGLDI)^53^ and a derivative of this NES with a tandem duplication of the export signal and the strong nuclear localization signal (NLS) of SV40 antigen (sequence: PKKKRKVG) were used to generate *ubp2-GFP-NES*, *ubp12-GFP-NES*, *ubp15-mRFP-NES*, and *ubp15-mRFP-NLS* forced localization mutants. Both the NLS (AAAPKKKRKVG) and the NES sequence (AAALALKLAGLNI) have been previously described^54^,^55^,^56^. For the generation of the forced localization mutants, the GFP-NES, mRFP-NES, and mRFP-NLS sequences were directly introduced at the 3′ end of the ORF of each *ubp* gene by PCR-mediated one-step gene replacement^52^. Plasmid vectors encoding GFP- or mRFP-NES and GFP- or mRFP-NLS forced localization cassettes will be described elsewhere.

### Immunoprecipitation and Western blot analysis

#### Protein extract preparation for Western analysis

TCA cell extracts were prepared and analysed as previously described for *S. cerevisiae*^11^,^57^,^58^. SDS-PAGE gels at 15%, 12%, 10%, and 8% were used for the detection of tubulin (15%); PCNA, PCNA-FLAG (12% and 10%), and Ubp16-myc (10%); and Ubp2-FLAG, Ubp2-myc, Ubp12-myc, and Ubp15-myc (8%). Whole-cell extracts for Western blotting were also obtained in HB buffer as previously described^48^.

#### Western blotting

Protein extracts and immunoprecipitates were electrophoresed using SDS-polyacrylamide gels ranging from 8 to 15%. For Western blots, 40–80 μ g of total protein extracts from each sample were blotted onto nitrocellulose, and proteins were detected using a characterized anti-PCNA affinity-purified polyclonal antibody (1:1500; a generous gift from Dr. Alan Lehmann). Tubulin was used as a loading control and detected with mouse TAT1 antitubulin monoclonal antibody (1:500). Antisera allowing the detection of PCNA and tubulin have been previously described^20^,^59^. We also used the anti-FLAG monoclonal antibody (1:3000) and the anti-Myc monoclonal antibody (1:3000). For all antibodies mentioned, goat anti-rabbit (1:3500) or goat anti-mouse (1:2000) antibodies conjugated to horseradish peroxidase (GE Healthcare, Chalfont St. Giles, United Kingdom) were used as secondary antibodies. Immunoblots were developed using Western Blotting Luminol Reagent (Santa Cruz Biotechnology, Santa Cruz, CA) or SuperSignal (Pierce Chemical, Rockford, IL) as required.

#### Protein extract preparation for immunoprecipitations

Soluble protein extracts were prepared basically as previously described^11^,^49^. Cells were collected, washed, and lysed in HB2T-lysis buffer using glass beads. The lysis buffer contained 60 mM β -glycerophosphate, 15 mM p-nitrophenylphosphate, 25 mM 4-morpholinepropanesulfonic acid (pH 7.2), 15 mM MgCl_2_, 15 mM EGTA, 1 mM dithiothreitol, 0.1 mM sodium orthovanadate, 2% Triton X-100, 1 mM phenylmethylsulfonyl fluoride, and 20 mg/ml leupeptin and aprotinin. The glass beads were washed with 500 μ l of HB2T, and the supernatant was recovered. For the immunoprecipitation studies or for the *in vitro* deubiquitylation assays, cell lysates (2–4 mg of protein) were incubated with the corresponding antibodies (2 μ g/mg extract) and protein G Dynabeads (Invitrogen) for 3 h at 4 °C. Immunoprecipitates were collected by centrifugation, washed 2–5 times with lysis buffer, and subjected either to SDS-PAGE electrophoresis or *in vitro* analysis. Protein concentrations were measured using the BCA assay kit (Pierce).

### *In vitro* deubiquitylation assays

PCNA-FLAG was efficiently immunoprecipitated from *ubp12-NES ubp15-NES* Δ *ubp16,* Δ *ubp2 ubp12-NES ubp15-NES* Δ *ubp16*, or *ubp12-NES ubp15-NES* Δ *ubp16* Δ *rad8* (*pcn1-FLAG*) strains (as indicated) after 2 hours in 20 mM HU. Ubp2-myc_13_, Ubp12-myc_13_, Ubp15-myc_13_, and Ubp16-myc_13_ were immunoprecipitated from asynchronous cultures of (*ubp2* or *ubp12* or *ubp15* or *ubp16*) myc_13_-tagged strains (we found that Ubp2, Ubp12, Ubp15, and Ubp16 are active throughout the cell cycle). The immunoprecipitations were washed two times with lysis buffer and then twice with DUB buffer (60 mM HEPES at pH 7.6, 5 mM MgCl_2_, 4% glycerol). Beads were incubated overnight at 30 °C. As a negative control, we used inactive Usp2, Usp12, Usp15, and Usp16 bound to ubiquitin vinyl sulfone (UbVS) DUB activity probe. This probe covalently captures active DUB enzymes and therefore acts as a potent and irreversible inhibitor of DUBs through the covalent modification of the active site, as previously described^60^. The UbVS probe was used as suggested by the manufacturer (Enzo Life Sciences). In some cases, a standard GST-tagged Ubp16 and Ubp16^CS^ protein expression and purification protocol was followed.

### MMS and drug sensitivity assays

Exponentially growing cells were counted and serially diluted in YES media. Tenfold dilutions of equal numbers of cells were used. Seven microliters of each dilution was spotted onto YES plates or YES plates containing different concentrations of MMS, HU, or 4-NQO (as indicated), incubated at 30 °C, and scanned. Plates were incubated for 2 to 4 days at 30 °C and scanned. MMS and 4-NQO plates were always freshly prepared.

### UV Sensitivity Assays

Cells were grown to logarithmic phase in YES media at 30 °C. Cultured cells were washed in fresh media, sonicated to disperse clumpy cells, and resuspended to a density of 2 ×  10^6^ cells per ml. Tenfold dilutions of these cells were spotted (7 μ l) onto YES plates. The plates were UV irradiated (as indicated) and incubated in the dark for 3 to 4 days at 30 °C and scanned.

## Additional Information

**How to cite this article**: Álvarez, V. *et al.* Orderly progression through S-phase requires dynamic ubiquitylation and deubiquitylation of PCNA. *Sci. Rep.* **6**, 25513; doi: 10.1038/srep25513 (2016).

## Supplementary Material

Supplementary Information

## References

[b1] Mailand N., Gibbs-Seymour I. & Bekker-Jensen S. Regulation of PCNA-protein interactions for genome stability. Nat. Rev. Mol. Cell Biol. 14, 269–282 (2013). 23594953 10.1038/nrm3562

[b2] Bergink S. & Jentsch S. Principles of ubiquitin and SUMO modifications in DNA repair. Nature 458, 461–467 (2009). 19325626 10.1038/nature07963

[b3] Ulrich H. D. Regulating post-translational modifications of the eukaryotic replication clamp PCNA. DNA Repair 8, 461–469 (2009). 19217833 10.1016/j.dnarep.2009.01.006

[b4] Andersen P. L., Xu F. & Xiao W. Eukaryotic DNA damage tolerance and translesion synthesis through covalent modifications of PCNA. Cell Res. 18, 162–173 (2008). 18157158 10.1038/cr.2007.114

[b5] Chang D. J. & Cimprich K. A. DNA damage tolerance: when it’s OK to make mistakes. Nat. Chem. Biol. 5, 82–90 (2009). 19148176 10.1038/nchembio.139 PMC2663399

[b6] Branzei D. & Foiani M. Maintaining genome stability at the replication fork. Nat. Rev. Mol. Cell Biol. 11, 208–219 (2010). 20177396 10.1038/nrm2852

[b7] Gallego-Sánchez A., Conde F., San Segundo P. & Bueno A. Control of PCNA deubiquitylation in yeast. Biochem. Soc. Trans. 38, 104–109 (2010). 20074044 10.1042/BST0380104

[b8] Ulrich H. D. & Takahashi T. Readers of PCNA modifications. Chromosoma 122, 259–274 (2013). 23580141 10.1007/s00412-013-0410-4 PMC3714560

[b9] Saugar I., Ortiz-Bazán M. Á. & Tercero J. A. Tolerating DNA damage during eukaryotic chromosome replication. Exp. Cell Res. 329, 170–177 (2014). 25038291 10.1016/j.yexcr.2014.07.009

[b10] Huang T. T. *et al.* Regulation of monoubiquitinated PCNA by DUB autocleavage. Nat. Cell Biol. 8, 339–347 (2006). 16531995 10.1038/ncb1378

[b11] Gallego-Sánchez A., Andrés S., Conde F., San-Segundo P. A. & Bueno A. Reversal of PCNA ubiquitylation by Ubp10 in Saccharomyces cerevisiae. Plos Genet. 8, e1002826 (2012). 22829782 10.1371/journal.pgen.1002826 PMC3400564

[b12] Park J. M. *et al.* Modification of PCNA by ISG15 plays a crucial role in termination of error-prone translesion DNA synthesis. Mol. Cell 54, 626–638 (2014). 24768535 10.1016/j.molcel.2014.03.031

[b13] Niimi A. *et al.* Regulation of proliferating cell nuclear antigen ubiquitination in mammalian cells. Proc. Natl. Acad. Sci. USA 105, 16125–16130 (2008). 18845679 10.1073/pnas.0802727105 PMC2571029

[b14] Oestergaard V. H. *et al.* Deubiquitination of FANCD2 is required for DNA crosslink repair. Mol. Cell 28, 798–809 (2007). 18082605 10.1016/j.molcel.2007.09.020 PMC2148256

[b15] Brown S., Niimi A. & Lehmann A. R. Ubiquitination and deubiquitination of PCNA in response to stalling of the replication fork. Cell Cycle 8, 689–692 (2009). 19221475 10.4161/cc.8.5.7707

[b16] Kannouche P. L., Wing J. & Lehmann A. R. Interaction of human DNA polymerase eta with monoubiquitinated PCNA: a possible mechanism for the polymerase switch in response to DNA damage. Mol. Cell 14, 491–500 (2004). 15149598 10.1016/s1097-2765(04)00259-x

[b17] Kannouche P. L. & Lehmann A. R. Ubiquitination of PCNA and the polymerase switch in human cells. Cell Cycle 3, 1011–1013 (2004). 15280666

[b18] Kim J. M. *et al.* Inactivation of murine Usp1 results in genomic instability and a Fanconi anemia phenotype. Dev. Cell 16, 314–320 (2009). 19217432 10.1016/j.devcel.2009.01.001 PMC3134285

[b19] Kouranti I. *et al.* A global census of fission yeast deubiquitinating enzyme localization and interaction networks reveals distinct compartmentalization profiles and overlapping functions in endocytosis and polarity. Plos Biol. 8, e1000471 (2010). 20838651 10.1371/journal.pbio.1000471 PMC2935449

[b20] Frampton J. *et al.* Postreplication repair and PCNA modification in Schizosaccharomyces pombe. Mol. Biol. Cell 17, 2976–2985 (2006). 16641370 10.1091/mbc.E05-11-1008 PMC1483034

[b21] Davies A. A., Huttner D., Daigaku Y., Chen S. & Ulrich H. D. Activation of ubiquitin-dependent DNA damage bypass is mediated by replication protein a. Mol. Cell 29, 625–636 (2008). 18342608 10.1016/j.molcel.2007.12.016 PMC2507760

[b22] Gallego-Sánchez A., Ufano S., Andrés S. & Bueno A. Analysis of the tolerance to DNA alkylating damage in MEC1 and RAD53 checkpoint mutants of Saccharomyces cerevisiae. Plos One 8, e81108 (2013). 24260543 10.1371/journal.pone.0081108 PMC3834268

[b23] Silva G. M., Finley D. & Vogel C. K63 polyubiquitination is a new modulator of the oxidative stress response. Nat. Struct. Mol. Biol. 22, 116–123 (2015). 25622294 10.1038/nsmb.2955 PMC4318705

[b24] Lee J.-G., Baek K., Soetandyo N. & Ye Y. Reversible inactivation of deubiquitinases by reactive oxygen species *in vitro* and in cells. Nature Communications 4, 1568 (2013). 10.1038/ncomms2532 PMC3615374 23463011

[b25] Prakash L. Characterization of postreplication repair in Saccharomyces cerevisiae and effects of rad6, rad18, rev3 and rad52 mutations. Mol. Gen. Genet. 184, 471–478 (1981). 7038396 10.1007/BF00352525

[b26] Gangavarapu V., Prakash S. & Prakash L. Requirement of RAD52 group genes for postreplication repair of UV-damaged DNA in Saccharomyces cerevisiae. Mol. Cell. Biol. 27, 7758–7764 (2007). 17785441 10.1128/MCB.01331-07 PMC2169055

[b27] Lambert S. *et al.* Homologous recombination restarts blocked replication forks at the expense of genome rearrangements by template exchange. Mol. Cell 39, 346–359 (2010). 20705238 10.1016/j.molcel.2010.07.015

[b28] González-Prieto R., Muñoz-Cabello A. M., Cabello-Lobato M. J. & Prado F. Rad51 replication fork recruitment is required for DNA damage tolerance. EMBO J. 32, 1307–1321 (2013). 23563117 10.1038/emboj.2013.73 PMC3642682

[b29] Coulon S. *et al.* Rad8Rad5/Mms2-Ubc13 ubiquitin ligase complex controls translesion synthesis in fission yeast. EMBO J. 29, 2048–2058 (2010). 20453833 10.1038/emboj.2010.87 PMC2892369

[b30] Hoege C., Pfander B., Moldovan G.-L., Pyrowolakis G. & Jentsch S. RAD6-dependent DNA repair is linked to modification of PCNA by ubiquitin and SUMO. Nature 419, 135–141 (2002). 12226657 10.1038/nature00991

[b31] Stelter P. & Ulrich H. D. Control of spontaneous and damage-induced mutagenesis by SUMO and ubiquitin conjugation. Nature 425, 188–191 (2003). 12968183 10.1038/nature01965

[b32] Haracska L., Torres-Ramos C. A., Johnson R. E., Prakash S. & Prakash L. Opposing effects of ubiquitin conjugation and SUMO modification of PCNA on replicational bypass of DNA lesions in Saccharomyces cerevisiae. Mol. Cell. Biol. 24, 4267–4274 (2004). 15121847 10.1128/MCB.24.10.4267-4274.2004 PMC400445

[b33] Watanabe K. *et al.* Rad18 guides poleta to replication stalling sites through physical interaction and PCNA monoubiquitination. EMBO J. 23, 3886–3896 (2004). 15359278 10.1038/sj.emboj.7600383 PMC522788

[b34] Zhuang Z. *et al.* Regulation of polymerase exchange between Poleta and Poldelta by monoubiquitination of PCNA and the movement of DNA polymerase holoenzyme. Proc. Natl. Acad. Sci. USA 105, 5361–5366 (2008). 18385374 10.1073/pnas.0801310105 PMC2291123

[b35] Mosbech A. *et al.* DVC1 (C1orf124) is a DNA damage-targeting p97 adaptor that promotes ubiquitin-dependent responses to replication blocks. Nat. Struct. Mol. Biol. 19, 1084–1092 (2012). 23042605 10.1038/nsmb.2395

[b36] Lou K. P. *et al.* Systems-wide analysis of ubiquitylation dynamics reveals a key role for PAF15 ubiquitylation in DNA-damage bypass. Nat. Cell Biol. 14, 1089–1098 (2012). 23000965 10.1038/ncb2579

[b37] Prakash S., Johnson R. E. & Prakash L. Eukaryotic translesion synthesis DNA polymerases: specificity of structure and function. Annu. Rev. Biochem. 74, 317–353 (2005). 15952890 10.1146/annurev.biochem.74.082803.133250

[b38] Branzei D. & Foiani M. Regulation of DNA repair throughout the cell cycle. Nat. Rev. Mol. Cell Biol. 9, 297–308 (2008). 18285803 10.1038/nrm2351

[b39] Giannattasio M. *et al.* Visualization of recombination-mediated damage bypass by template switching. Nat. Struct. Mol. Biol. 21, 884–892 (2014). 25195051 10.1038/nsmb.2888 PMC4189914

[b40] Fumasoni M., Zwicky K., Vanoli F., Lopes M. & Branzei D. Error-free DNA damage tolerance and sister chromatid proximity during DNA replication rely on the Polα /Primase/Ctf4 Complex. Mol. Cell 57, 812–823 (2015). 25661486 10.1016/j.molcel.2014.12.038 PMC4352764

[b41] Indiani C., Langston L. D., Yurieva O., Goodman M. F. & O’donnell M. Translesion DNA polymerases remodel the replisome and alter the speed of the replicative helicase. Proc. Natl. Acad. Sci. USA 106, 6031–6038 (2009). 19279203 10.1073/pnas.0901403106 PMC2654394

[b42] Li J., Yuan Z. & Zhang Z. The cellular robustness by genetic redundancy in budding yeast. Plos Genet. 6, e1001187 (2010). 21079672 10.1371/journal.pgen.1001187 PMC2973813

[b43] Daigaku Y., Davies A. A. & Ulrich H. D. Ubiquitin-dependent DNA damage bypass is separable from genome replication. Nature 465, 951–955 (2010). 20453836 10.1038/nature09097 PMC2888004

[b44] Karras G. I. & Jentsch S. The RAD6 DNA damage tolerance pathway operates uncoupled from the replication fork and is functional beyond S phase. Cell 141, 255–267 (2010). 20403322 10.1016/j.cell.2010.02.028

[b45] Ortiz-Bazán M. Á. *et al.* Rad5 Plays a Major Role in the Cellular Response to DNA Damage during Chromosome Replication. Cell Reports (2014), doi: 10.1016/j.celrep.2014.09.005. 25310987

[b46] Moreno S., Klar A. & Nurse P. Molecular genetic analysis of fission yeast Schizosaccharomyces pombe. Meth Enzymol 194, 795–823 (1991). 2005825 10.1016/0076-6879(91)94059-l

[b47] Forsburg S. L. & Rhind N. Basic methods for fission yeast. Yeast 23, 173–183 (2006). 16498704 10.1002/yea.1347 PMC5074380

[b48] Cueille N. *et al.* Flp1, a fission yeast orthologue of the s. cerevisiae CDC14 gene, is not required for cyclin degradation or rum1p stabilisation at the end of mitosis. J. Cell Sci. 114, 2649–2664 (2001). 11683392 10.1242/jcs.114.14.2649

[b49] Díaz-Cuervo H. & Bueno A. Cds1 controls the release of Cdc14-like phosphatase Flp1 from the nucleolus to drive full activation of the checkpoint response to replication stress in fission yeast. Mol. Biol. Cell 19, 2488–2499 (2008). 18385517 10.1091/mbc.E07-08-0737 PMC2397296

[b50] Esteban V. *et al.* A role for the Cdc14-family phosphatase Flp1p at the end of the cell cycle in controlling the rapid degradation of the mitotic inducer Cdc25p in fission yeast. J. Cell. Sci. 117, 2461–2468 (2004). 15128870 10.1242/jcs.01107

[b51] Esteban V., Sacristán M., Andrés S. & Bueno A. The Flp1/Clp1 phosphatase cooperates with HECT-type Pub1/2 protein-ubiquitin ligases in Schizosaccharomyces pombe. Cell Cycle 7, 1269–1276 (2008). 18418059 10.4161/cc.7.9.5947

[b52] Longtine M. S. *et al.* Additional modules for versatile and economical PCR-based gene deletion and modification in Saccharomyces cerevisiae. Yeast 14, 953–961 (1998). 9717241 10.1002/(SICI)1097-0061(199807)14:10<953::AID-YEA293>3.0.CO;2-U

[b53] Wen W., Meinkoth J. L., Tsien R. Y. & Taylor S. S. Identification of a signal for rapid export of proteins from the nucleus. Cell 82, 463–473 (1995). 7634336 10.1016/0092-8674(95)90435-2

[b54] Edgington N. P. & Futcher B. Relationship between the function and the location of G1 cyclins in S. cerevisiae. J. Cell Sci. 114, 4599–4611 (2001). 11792824 10.1242/jcs.114.24.4599

[b55] Hodel A. E. *et al.* Nuclear localization signal receptor affinity correlates with *in vivo* localization in Saccharomyces cerevisiae. J. Biol. Chem. 281, 23545–23556 (2006). 16785238 10.1074/jbc.M601718200

[b56] Rossio V. & Yoshida S. Spatial regulation of Cdc55-PP2A by Zds1/Zds2 controls mitotic entry and mitotic exit in budding yeast. J. Cell. Biol. 193, 445–454 (2011). 21536748 10.1083/jcb.201101134 PMC3087000

[b57] Longhese M. P. *et al.* The novel DNA damage checkpoint protein ddc1p is phosphorylated periodically during the cell cycle and in response to DNA damage in budding yeast. EMBO J. 16, 5216–5226 (1997). 9311982 10.1093/emboj/16.17.5216 PMC1170154

[b58] Cordon-Preciado V., Ufano S. & Bueno A. Limiting amounts of budding yeast Rad53 S-phase checkpoint activity results in increased resistance to DNA alkylation damage. Nucleic Acids Res. 34, 5852–5862 (2006). 17062626 10.1093/nar/gkl741 PMC1635317

[b59] Bähler J. & Pringle J. R. Pom1p, a fission yeast protein kinase that provides positional information for both polarized growth and cytokinesis. Genes Dev. 12, 1356–1370 (1998). 9573052 10.1101/gad.12.9.1356 PMC316787

[b60] Borodovsky A. *et al.* A novel active site-directed probe specific for deubiquitylating enzymes reveals proteasome association of USP14. EMBO J. 20, 5187–5196 (2001). 11566882 10.1093/emboj/20.18.5187 PMC125629

