# Supplementary material for: Orderly progression through S-phase requires dynamic ubiquitylation and deubiquitylation of PCNA
Source: Sci Rep. 2016 May 6;6:25513. doi: 10.1038/srep25513 (PMC4858703; doi:10.1038/srep25513)
Supplement: Supplementary Information [file srep25513-s1.pdf]

Supplementary Information

Orderly progression through S-phase requires dynamic ubiquitylation and deubiquitylation of PCNA

Vanesa Álvarez, Laura Viñas, Alfonso Gallego-Sánchez, Sonia Andrés, María Sacristán and Avelino Bueno

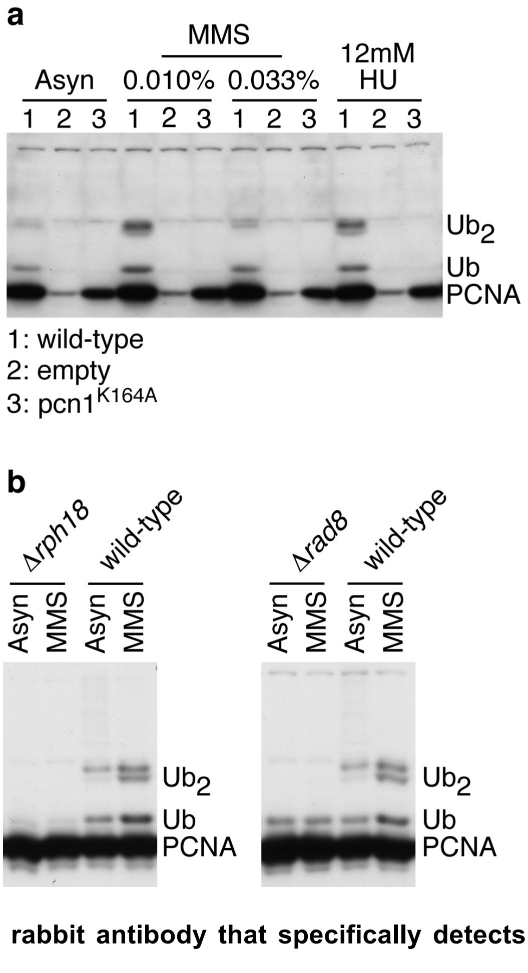

Figure S1. A polyclonal rabbit antibody that specifically detects PCNA forms in fission yeast cell extracts.

Immunoblot analysis with affinity purified rabbit α-PCNA antibody of TCA-protein extracts from wild-type, *pcn1*^{K164R} (unable to ubiquitylate PCNA) (**a**), *Δrhp18* (unable to ubiquitylate PCNA) (**b**, left panel) and *Δrad8* (unable to di-ubiquitylate or poly-ubiquitylate PCNA) (**b**, right panel) cells growing exponentially (Asyn), treated 3 hours with MMS (0.01% or 0.033%, as indicated) or treated 3 hours with HU and resolved in 12% polyacrylamide gels.

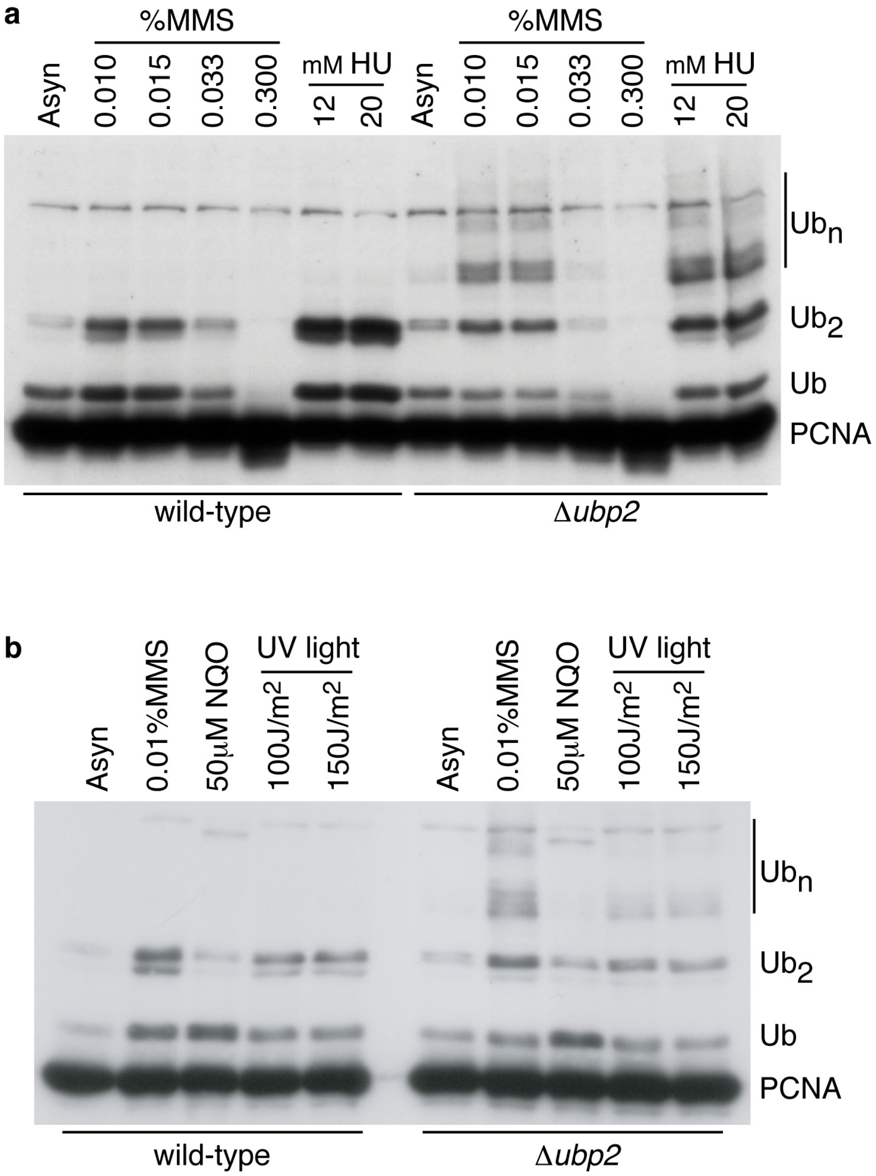

Figure S2. Cells lacking *ubp2*$^+$ accumulate poly-ubiquitylated PCNA in response to DNA damage and replicative stress.

Immunodetection of ubiquitylated forms of PCNA in wild-type and *Δubp2* TCA-cell extracts to show that *ubp2* mutant cells accumulate K164 mono-ub, di-ub and poly-ubPCNA forms. **a**. Immunodetection of PCNA forms in wild-type and *Δubp2* fission yeast cells treated 3 hours with methyl methanesulfonate (0.010% MMS, 0.015% MMS, 0.033% MMS and 0.3% MMS) or hydroxyurea (12 mM HU and 20 mM HU). **b**. Immunodetection of PCNA forms in wild-type and *Δubp2* fission yeast cells treated 3 hours with 0.010% MMS and 50mM 4-NQO or exposed to 100 J/m2 and 150 J/m2 UV-light (as indicated).

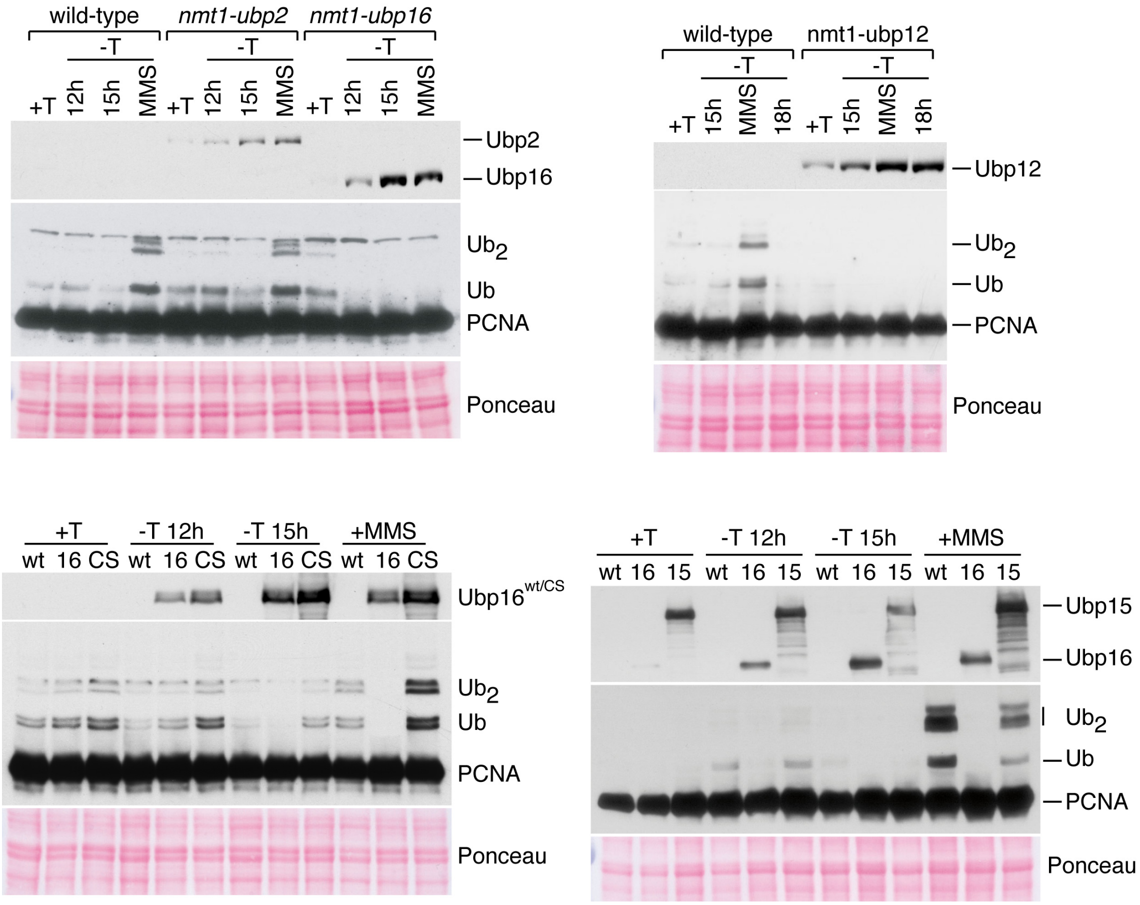

Figure S3. Analysis PCNA ubiquitylation in *nmt1*-regulated overexpressing *ubp2*$^{+}$*, ubp12*$^{+}$*, ubp15*$^{+}$*, and ubp16*$^{+}$ fission yeast cells.

Increased expression of *ubp12*$^{+}$ or *ubp16*$^{+}$ prevented the accumulation of ubiquitylated PCNA, while overexpression of *ubp15*$^{+}$ diminished the amount of PCNA modified forms. However, *ubp2*$^{+}$ overexpressing cells accumulated normal levels of mono- and di-ubiquitylated PCNA forms. Expression of the indicated *ubp* genes was induced by incubation in media without thiamine (-T) at 32ºC. Samples were removed at 12, 15, 18 hours untreated or MMS-treated intervals (as indicated), and processed for Western blot analysis. A negative control (+T) of cells repressed in the presence of thiamine is shown. An additional negative control, a catalytically inactive Ubp16^{C134S} mutant form (CS or Ubp16^{CS}) is also shown.

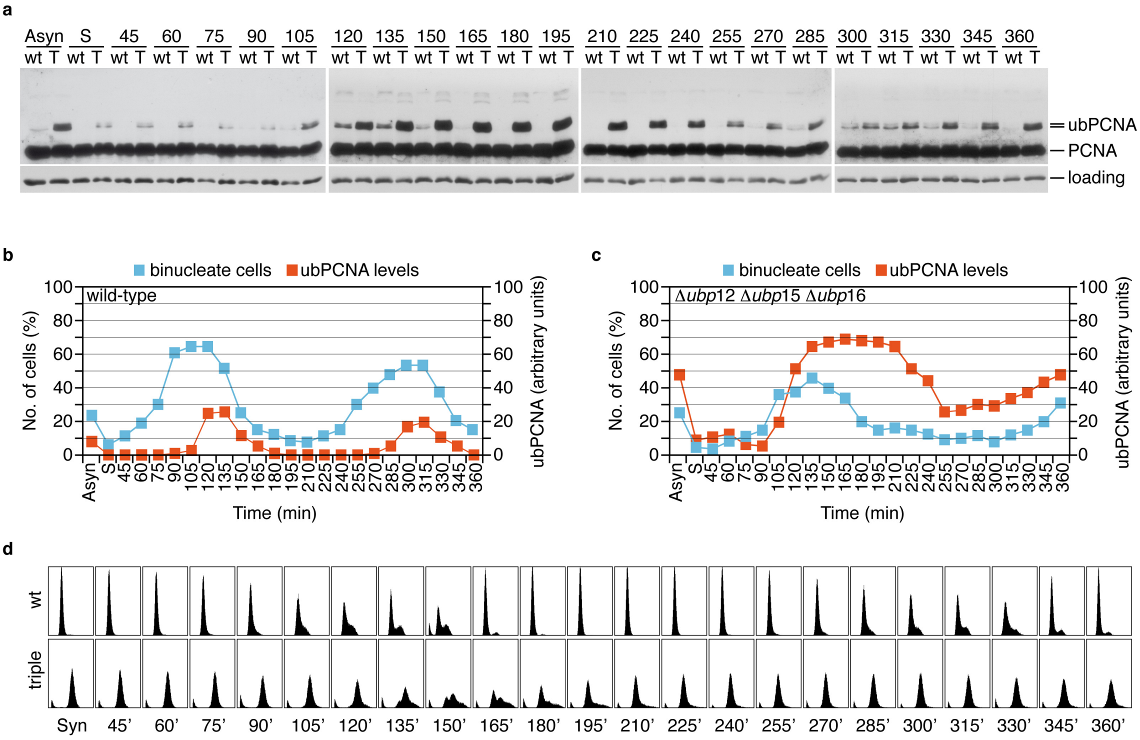

Figure S4. Analysis of PCNA ubiquitylation and cell cycle progression in wild-type and *Δubp12 Δubp15 Δubp16* triple mutant strains synchronized by elutriation.

Cells of wild-type (wt) and *Δubp12 Δubp15 Δubp16* (T) strains synchronized by elutriation in G2 (S) were released at 23°C in fresh media. **a**. Samples were taken at indicated intervals and processed for the immunodetection of modified PCNA forms, PCNA, and alpha-tubulin (loading). Cell cycle distribution was determined by the microscopic measurement of the percentage of binucleated cells in wild-type (**b**) and *Δubp12 Δubp15 Δubp16* (**c**) cultures and by the FACS analysis of DNA content in cells stained with propidium iodide in **d**. Mono-ubiquitylated PCNA was quantified, normalized, and plotted in **b** and **c**. Note that in wild-type cells mono-ubiquitylated PCNA was detected immediately after the peak of mitosis (in *S. pombe* septation occurs in early S phase, and the S phase coincides with PCNA ubiquitylation).

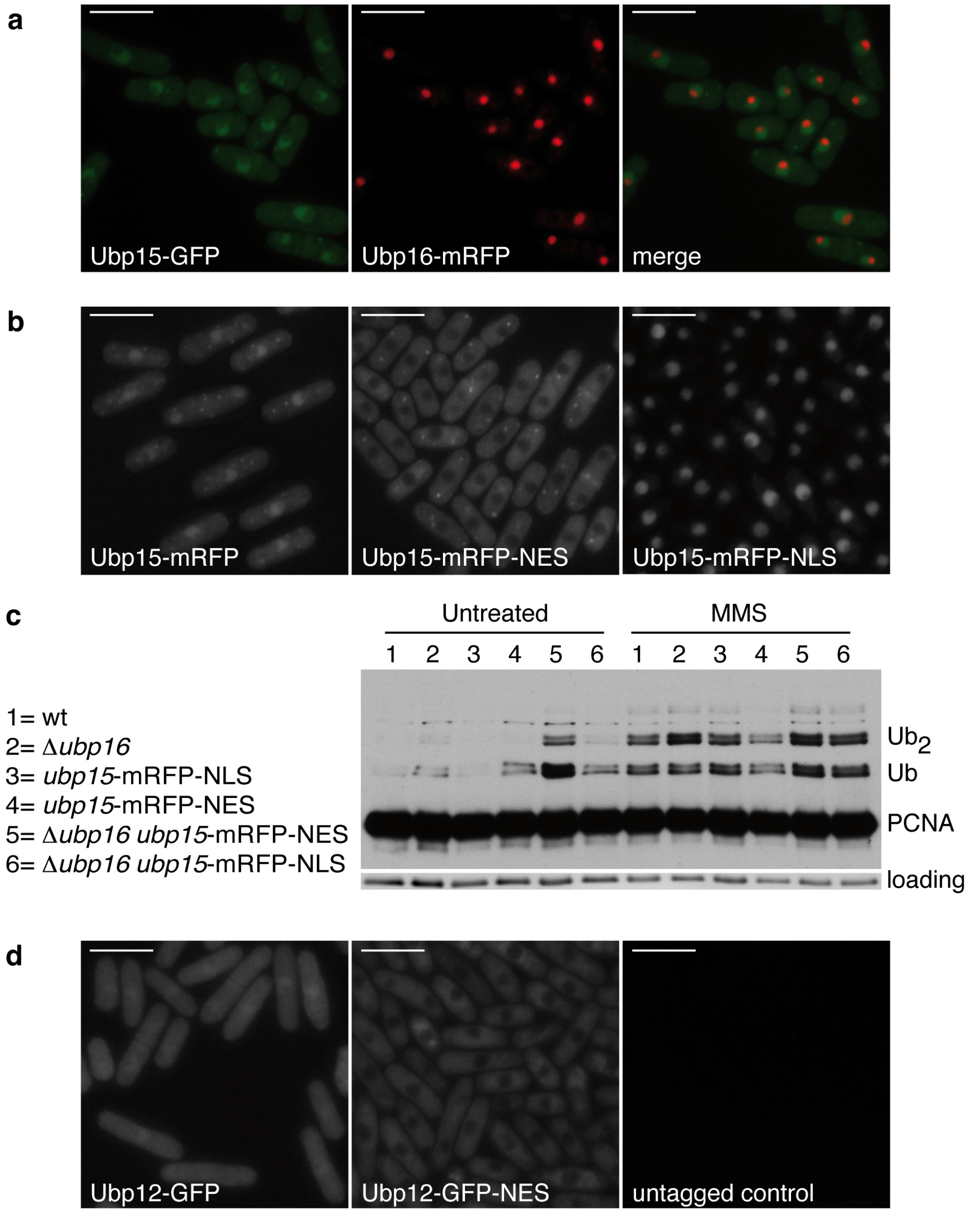

Figure S5. Nuclear exclusion of Ubp15 mimics the defects associated with the deletion of *ubp15*$^{+}$.

a. Left panel, Ubp15-GFP localizes to both the nucleus and specific cytoplasmatic structures (as previously described, Kouranti et al., 2010). Central panel, Ubp16-mRFP shows a strong homogeneous nucleolar pattern. Right panel, combined left and central images. **b**. Left panel, Ubp15-mRFP localizes to the nucleus and specific cytoplasmatic structures. Central panel, addition of a strong nuclear export signal (NES, see Methods) to Ubp15-mRFP efficiently excludes this ubiquitin protease from the nucleus. Right panel, a Ubp15-mRFP-NLS protein, fused to the robust SV40 nuclear localization signal, is constitutively nuclear. Bars, 10μm. **c**. Immunodetection of PCNA forms in the indicated fission yeast strains untreated or treated 90 minutes with 0.010% MMS. Note the accumulation of mono-ubiquitylated PCNA in untreated *ubp15*-mRFP-NES cells (lane 4) and the strong accumulation of mono-ubiquitylated PCNA in untreated *Δubp16 ubp15*-mRFP-NES double mutant cells (lane 5). Together, *in vivo* Ubp15-mRFP-NLS subcellular localization and Western blot observations are consistent with a role for Ubp15 in deubiquitylating ubPCNA in the non-nucleolar fraction of the nucleus. **d**. Left panel, Immunofluorescence microscopy image of Ubp12-GFP showing that it localizes uniformly diffuse to both the cytoplasm and nucleus (as previously described, Kouranti et al., 2010). Central panel, a strong nuclear export signal fused to C-terminus of Ubp12 excludes this ubiquitin protease from the nucleus. Right panel, untagged control.

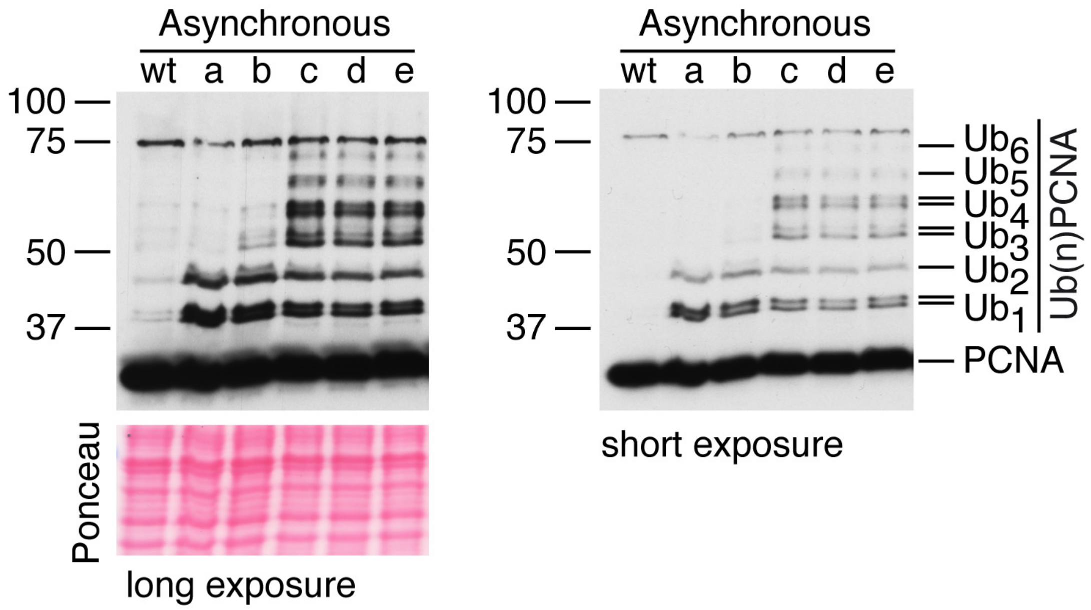

Figure S6. Comparative analysis of modified PCNA forms in *Δubp2, ubp2-NES, ubp12-NES, ubp15-NES,* and *Δubp16* multiple mutant strains.

Accumulation of mono-, di-, and poly-ubiquitylated forms of wild-type PCNA (untagged) in *S.pombe* mutant cells defective in ubPCNA deubiquitylation. Cell extracts from wild-type (wt), *ubp12-NES ubp15-NES Δubp16* (a,b), and *Δubp2 ubp12-NES ubp15-NES Δubp16* (c,d,e) were resolved in 12% polyacrylamide gels and immunobloted with affinity purified rabbit α-PCNA antibody.

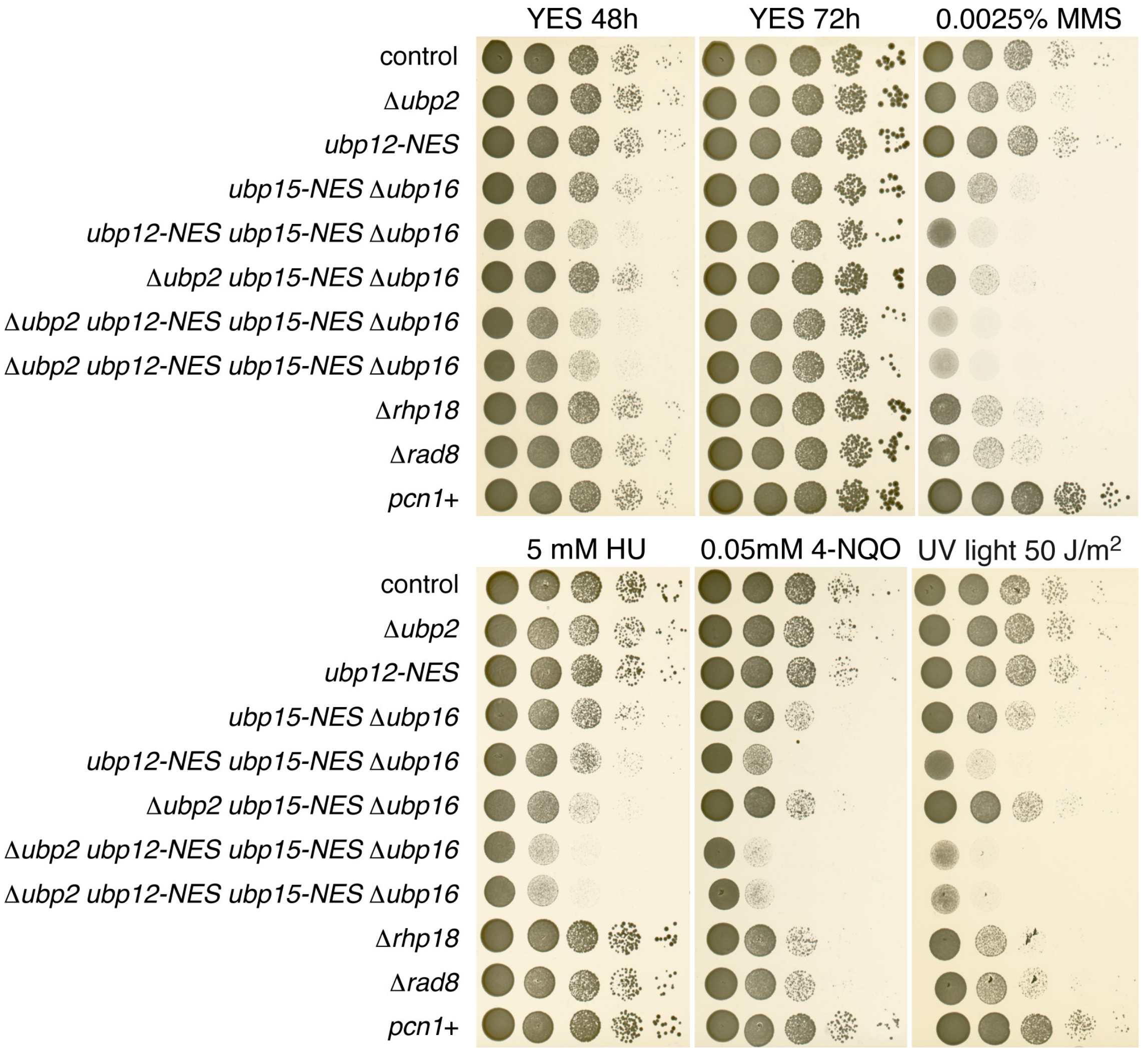

Figure S7. *S.pombe* cells lacking PCNA ubiquitin-specific proteases are hypersensitive to DNA damage and HU-induced replication stress.

Sensitivity assay of relevant *ubp2*$^{+}$, *ubp12*$^{+}$, *ubp15*$^{+}$ and *ubp16*$^{+}$ (simple and multiple) mutant alleles to HU and MMS-, 4-NQO- and UV-light-induced DNA damage. All the strains are *pcn1*-FLAG (except where indicated: *pcn1*$^{+}$). Tenfold (10-fold) serial dilution assays of cultures of the indicated strains exposed to sublethal concentrations of DNA replication inhibitor HU or DNA damaging agents.

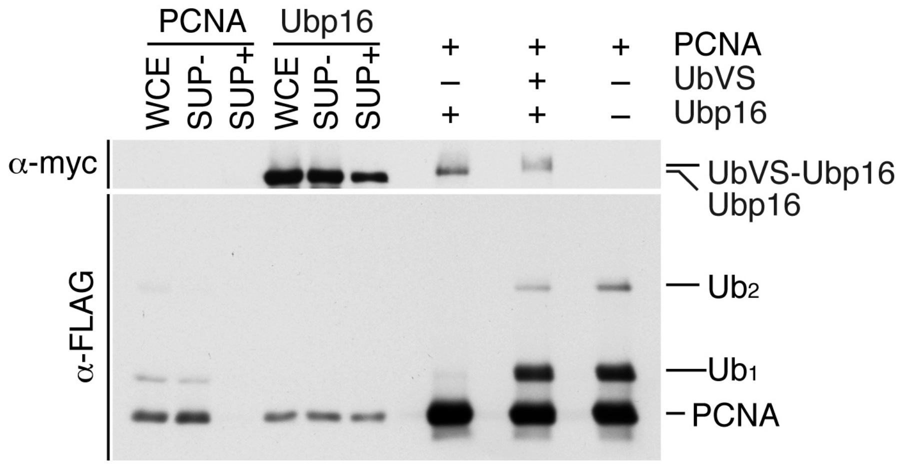

Figure S8. Ubp16 deubiquitylates mono- and di-ubiquitylated PCNA *in vitro*.

Mono- (Ub_1) and di-ubiquitylated (Ub_2) PCNA was obtained by immunoprecipitation with anti-FLAG antibody from a *ubp12-NES ubp15-NES Δubp16 pcn1-FLAG* strain, cells were pre-synchronized in early S-phase (3 hours in 20 mM HU). Immunoprecipitated samples were divided in three, and two of the aliquots were incubated with immunoprecipitated Ubp16-myc in the absence or in the presence of UbVS to inhibit any UBP activity (as described in the Methods section). The third aliquot served as a reference sample of the immunoprecipitated PCNA. PCNA deubiquitylation was detected by the distinctive SDS-PAGE gel mobility of the different PCNA forms. Whole cell extracts (WCE), depleted supernatants (SUP+) and non-depleted supernatants (SUP-) from PCNA-FLAG and Ubp16-myc strains are also shown.

a

	Untreated, (%) of cells			HU 4h, (%) of cells		
	1 focus	> 1 foci	no foci	1 focus	> 1 foci	no foci
wild-type	11.96±0.75	2.14±0.20	85.89±0.55	11.19±2.22	6.90±0.55	81.89±2.65
Δ*cds1*	16.84±2.94	12.90±2.06	70.26±5.01	14.92±2.54	72.23±4.29	12.83±6.84
ubp2-NES	11.69±2.28	2.14±0.73	86.16±3.02	24.64±5.80	26.59±13.52	48.77±7.55
ubp12-NES	9.64±1.36	1.42±0.50	88.93±0.85	10.46±2.85	7.30±3.83	82.23±2.05
ubp15-NES	13.95±3.40	2.75±1.71	83.28±4.34	7.83±2.55	6.80±1.77	85.35±2.20
Δ*ubp16*	11.23±1.99	2.49±1.21	86.27±3.21	5.59±2.16	4.39±1.80	90.01±1.90

b

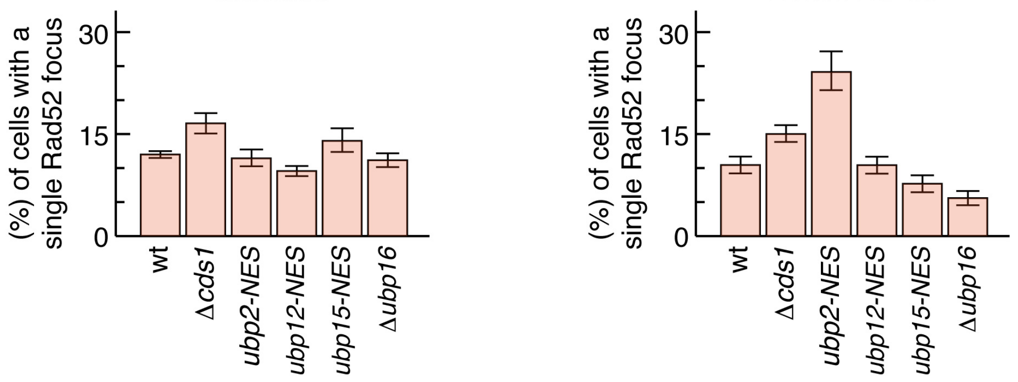

Figure S9. Rad52-YFP foci in *ubp2*, *ubp12*, *ubp15* and *ubp16* fission yeast single mutants deficient in ubPCNA deubiquitylation.

a. Quantification of nuclei containing single or multiple Rad52-foci in fission yeast strains in asynchronous exponentially growing cells (untreated) and after 4 hours of 20mM HU treatment. Control counts in checkpoint proficient wild-type cells and checkpoint mutants *Δcds1* are also shown. Results are representative of three independent experiments (to gain an estimate of error, where n is over 1200 for each sample). Standard deviations are shown in the table for each % of cells. **b.** Plots of the data regarding % of cells harbouring single Rad52 focus.

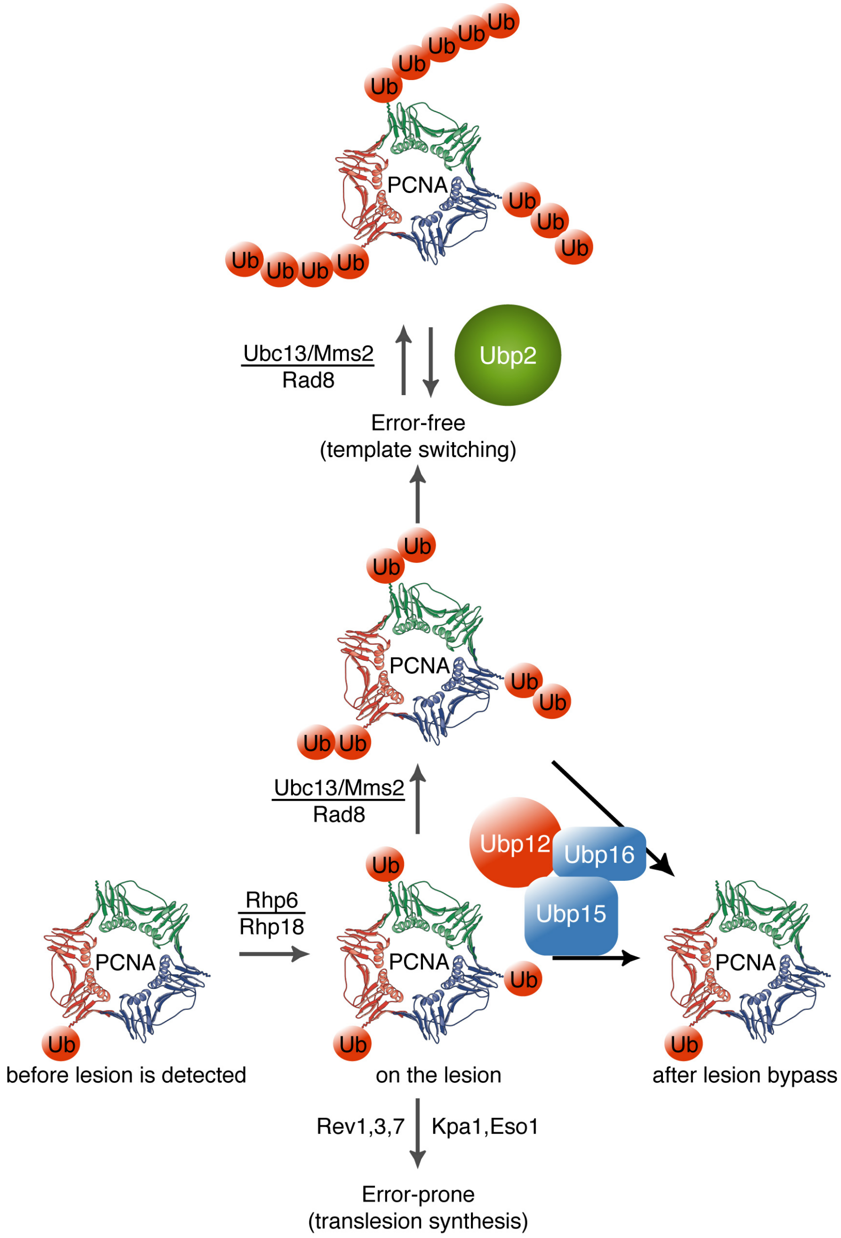

Figure S10. Working model for ubPCNA deubiquitylation in fission yeast.
Model for the role of Ubp2, Ubp12, Ubp15, and Ubp16 in the modulation of PCNA ubiquitylation in *S. pombe* cells. PCNA homotrimers progress with the replisome at replication forks. The detection of bulky lesions on DNA impedes fork progression and induces the Rhp6/Rhp18 mono-ubiquitylation of PCNA at lysine 164; therefore, it favours lesion bypass by enhancing ubPCNA-TLS DNA polymerase interaction or further ubPCNA (K63-linked) polyubiquitylation (by the Ubc13/Mms2/Rad8 ubiquitin ligase complex). After lesion bypass, Ubp15 and Ubp16 (likely with the help of Ubp12) deubiquitylate di- and mono-ubPCNA to allow the remodelling of the replisome by switching back to replicative DNA polymerases, thus resuming rapid and processive DNA replication fork progression. In some circumstances (i.e., oxidative DNA damage), di-ubiquitylated PCNA is further polyubiquitylated (likely by the same Ubc13/Mms2/Rad8 ubiquitin ligase). Ubp2 deubiquitylates these (long K63-linked) polyubiquitylated PCNA forms in a manner that results in a net gain of di-ubiquitylated PCNA. The unwanted step (the accumulation of ub_1-PCNA as an intermediate form in PCNA deubiquitylation) is bypassed by the ability of Ubp15 and Ubp16 proteases to deubiquitylate both mono- and di-ubiquitylated PCNA, thus acting as efficient substrate-specific DUBs for the sliding clamp.
